# Radiomics-Based Quality Control System for Automatic Cardiac Segmentation: A Feasibility Study

**DOI:** 10.3390/bioengineering10070791

**Published:** 2023-07-01

**Authors:** Qiming Liu, Qifan Lu, Yezi Chai, Zhengyu Tao, Qizhen Wu, Meng Jiang, Jun Pu

**Affiliations:** Department of Cardiology, Renji Hospital, Shanghai Jiao Tong University School of Medicine, Shanghai 200120, China; undomiel.lqm@gmail.com (Q.L.);

**Keywords:** radiomics, deep learning, quality control, cardiac magnetic resonance

## Abstract

Purpose: In the past decade, there has been a rapid increase in the development of automatic cardiac segmentation methods. However, the automatic quality control (QC) of these segmentation methods has received less attention. This study aims to address this gap by developing an automatic pipeline that incorporates DL-based cardiac segmentation and radiomics-based quality control. Methods: In the DL-based localization and segmentation part, the entire heart was first located and cropped. Then, the cropped images were further utilized for the segmentation of the right ventricle cavity (RVC), myocardium (MYO), and left ventricle cavity (LVC). As for the radiomics-based QC part, a training radiomics dataset was created with segmentation tasks of various quality. This dataset was used for feature extraction, selection, and QC model development. The model performance was then evaluated using both internal and external testing datasets. Results: In the internal testing dataset, the segmentation model demonstrated a great performance with a dice similarity coefficient (DSC) of 0.954 for whole heart segmentations. Images were then appropriately cropped to 160 × 160 pixels. The models also performed well for cardiac substructure segmentations. The DSC values were 0.863, 0.872, and 0.940 for RVC, MYO, and LVC for 2D masks and 0.928, 0.886, and 0.962 for RVC, MYO, and LVC for 3D masks with an attention-UNet. After feature selection with the radiomics dataset, we developed a series of models to predict the automatic segmentation quality and its DSC value for the RVC, MYO, and LVC structures. The mean absolute values for our best prediction models were 0.060, 0.032, and 0.021 for 2D segmentations and 0.027, 0.017, and 0.011 for 3D segmentations, respectively. Additionally, the radiomics-based classification models demonstrated a high negative detection rate of >0.85 in all 2D groups. In the external dataset, models showed similar results. Conclusions: We developed a pipeline including cardiac substructure segmentation and QC at both the slice (2D) and subject (3D) levels. Our results demonstrate that the radiomics method possesses great potential for the automatic QC of cardiac segmentation.

## 1. Introduction

Cardiac magnetic resonance (CMR) is currently the gold standard noninvasive imaging tool for the evaluation of heart function [1]. However, the foremost step in CMR analysis is precise segmentation. In addition to clinical experience, accurate parameter quantification is also essential for clinical decision making and risk stratification [2]. Unfortunately, manual segmentation is a time-consuming process which also introduces intra- or interoperator variability. Although automatic segmentation has advanced rapidly in recent years, human-level quality control (QC) of automatic segmentation is still mandatory for clinical purposes. This is particularly important for the segmentation of small objects, such as apical slices in CMR imaging or small nodules in lung CT scans [3]. More importantly, low-quality and inaccurate segmentation is hard to detect and may led to dramatic consequences. However, it is impractical to implement manual QC into an automatic pipeline. This highlights the urgent need for the development of an automatic QC system.

The purpose of the automatic segmentation tools is to lessen the burden for doctors and researchers, as well as to improve the stability and quality of segmentation. Additionally, these tools should provide a quality score for each automatic segmentation. Therefore, the absence of quality control is a limitation of automatic segmentation software, along with high expenses, patent restrictions, etc.

In the past decade, there has been an increasing amount of research on medical image QC systems. As a result, several automatic QC systems have been developed for medical image segmentation. Kohlberger developed a classifier to predict the error in segmentation methods without ground truth (GT) [4]; Alba developed an automatic QC system based on the RF-based detector, a 3D-SPASM segmentation algorithm, and an anatomically driven classifier [5]. Valindria proposed a reverse classification accuracy (RCA) method to predict the performance of a segmentation model on new data without GT [6], and the RCA method was further adopted by Robinson for cardiac segmentation [7]. More recently, deep learning (DL)-based prediction models have gained popularity. Fournel developed a DL-based automatic segmentation DSC prediction method and achieved good performances with various types of disease [8]. Li proposed a pixel-level and image-level quality assessment system [9]. All of the previous works have exhibited the importance of the QC system.

Radiomics has been developed for over a decade and has demonstrated impressive performances in various tasks. Additionally, radiomics features are calculated using precise equations, providing a significant advantage in terms of explainability. Furthermore, the computation load for radiomics methods is also significantly lower than those of DL methods. More importantly, CMR segmentation is an excellent imaging modality for radiomics analysis. This technique allows for the extraction of 2D features from single slice images as well as 3D features from reconstructed 3D images made up of 2D image stacks. The applications of radiomics have facilitated cardiovascular disease phenotyping [10], differential diagnosis [11], and prognosis prediction [12] etc. However, we observed that few studies have used radiomics methods as tools for QC. Maffei proposed a radiomics-based QC system for cardiac CT segmentation, but their models were quantitative (only predict whether a segmentation is clinically acceptable or not), and the feature number used in their study was tremendous (a total of 78,000 for 25 substructures) [13]. Although not in the field of cardiovascular disease, Sunoqrot showed that radiomics information could help to generate a quantitative quality score and facilitate the QC of prostate segmentation on T2 images [14]; Wootton and Sakai also used radiomics to detect errors in radiation therapy [15,16].

Based on current understanding and previous studies of radiomics, we hypothesize that radiomics features can aid in the quality control of the segmentation of CMR images [17]. The objective of this study is to establish a compatible pipeline that could incorporate DL-based segmentation and radiomics-based QC for CMR short-axis cine images. Our proposed pipeline has the following functions: (a) localizing, cropping, and segmenting the whole heart using DL; (b) detecting low-quality segmentations based on radiomics features (qualitative QC); (c) predicting DSC scores for automatic segmentation (quantitative QC); and (d) visualizing and analyzing the results.

## 2. Methods

**Figure 1:**
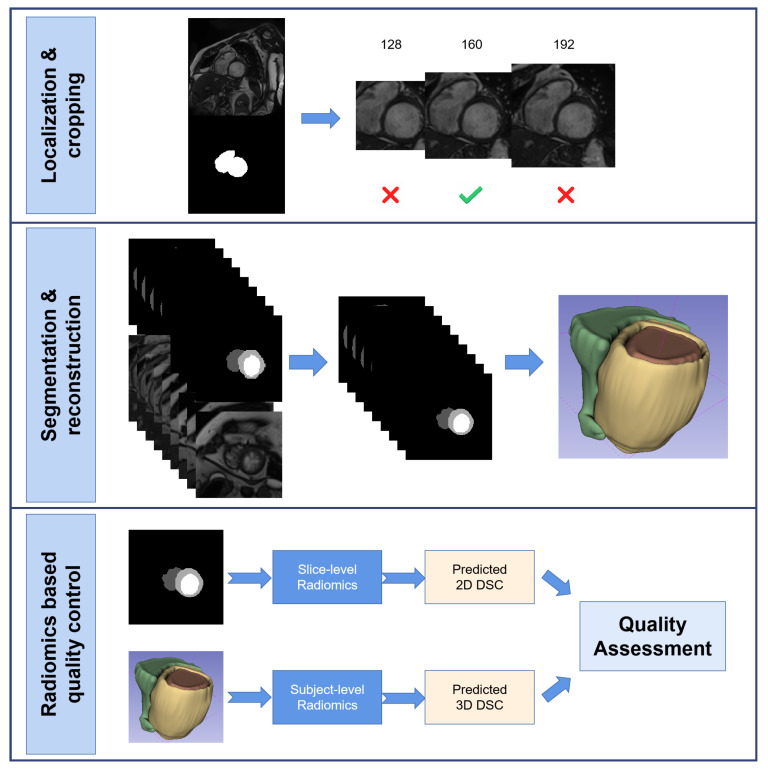
This is the flowchart of this study. It mainly contains three part, a DL-based whole heart localization and images cropping part; a DL-based anatomical structures segmentation part and a radiomics-based quality control part.

Figure 1 shows the flowchart of this study.

### 2.1. Study Population

In this study, data were obtained from a private dataset and two publicly available external datasets. The private dataset was obtained from August 2017 to December 2021 from Shanghai Renji Hospital (Renji-2021) and included hypertrophic cardiomyopathy (HCM), dilated cardiomyopathy (DCM), hypertensive heart disease (HHD), and healthy controls (HC). The first external dataset containing HCM, DCM, and HC was obtained from the 2017 Automated Cardiac Diagnosis Challenge (ACDC-2017) training dataset [18]. The second external dataset containing HCM, HHD, DCM, and HC was obtained from the 2020 M&M challenge (M&M-2020). The detailed inclusion and exclusion criteria for the Renji-2021 dataset were as follows.

The HCM inclusion criteria were (a) the genetic determination of an HCM mutation; (b) left ventricle hypertrophy (LVH) > 15 mm in the absence of known causes of hypertrophy [19]; and (c) hypertrophy in a recognizable pattern, i.e., apical-variant HCM.

The HHD inclusion criteria were (a) electrocardiograph (ECG) demonstration of a hypertrophic LV (maximal LV wall thickness > 11 mm or LV mass to body surface area > 115 g/m^2^ for men or >95 g/m^2^ for women) in the absence of other cardiac or systemic diseases [20] or (b) a diagnosis of arterial hypertension [21].

DCM is defined by the presence of left ventricular (LV) dilation and systolic dysfunction in the absence of abnormal loading conditions or coronary artery disease sufficient to cause LV systolic impairment [22].

The HC group consisted of healthy volunteers who demonstrated normal cardiac dimensions and volumes, normal cardiac function, and the absence of late gadolinium enhancement. None of the control subjects had a history of known cardiac disease, including cardiac surgery and interventions.

Exclusion criteria for all subjects were an established diagnosis of Fabry disease, cardiac amyloidosis, severe valvular disease, aortic stenosis, iron deposition, evidence of inflammatory processes in the myocardium or pericardium, history of ST-segment elevation myocardial infarction, and subjects experiencing activity of sufficient duration, intensity, and frequency to explain the abnormal LV wall thickness.

### 2.2. Image Acquisition

Renji-2021 dataset: The CMR examinations were performed with a 3T MRI scanner (Ingenia, Philips). A balanced steady-state free precision (SSFP) sequence with breath hold was used for cine imaging acquisition. The typical parameters were as follows: slice thickness = 6–8 mm, gap between slices = 6–10 mm, in-plane resolution = 0.8–1.2 mm × 0.8–1.2 mm, and number of cardiac phases = 30.

ACDC-2017 dataset: The acquisitions were obtained using a 1.5T scanner (Area, Siemens) and a 3T scanner (Trio Tim, Siemens). A conventional SSFP sequence with breath hold was used for cine imaging acquisition. The typical parameters were as follows: slice thickness = 5–8 mm, gap between slices = 5–10 mm, in-plane resolution = 1.37–1.68 mm × 1.37–1.68 mm, and number of cardiac phases = 28–40.

M&M-2020 dataset: In this dataset, CMR images were acquired with scanners from different vendors (Siemens, Philips, GE and Canon) with both 1.5T and 3.0T magnetic fields. The parameters were as follows: tTypical slice thickness = 9.2–10 mm, typical gap between slices = 10 mm, in-plane resolution = 0.85–1.45 mm × 0.85–1.45 mm, number of slices = 10–12, and number of frames = 25–30.

For all datasets, short-axis cine images covered LV and RV from the base to the apex.

### 2.3. Data Preparation

For all datasets, only end-diastole (ED) short-axis cine images were included in this study.

For the Renji dataset, images were exported in digital imaging and communications in medicine (DICOM) format.Foremost, patients’ information was de-identified. An experienced cardiologist (4 years of CMR experience) manually removed images of insufficient quality. Selected ED images were then converted to .nii format (same as for the ACDC-2017 and M&M-2020 datasets).

#### 2.3.1. Manual Segmentation

Manual segmentation results from the ACDC-2017 and M&M datasets were available from the ACDC challenge website https://www.creatis.insa-lyon.fr/Challenge/acdc/databases.html (accessed on 1 January 2023) and M&M challenge website https://www.ub.edu/mnms/ (accessed on 1 June 2023). An open-source software itk-snap (version 3.8.0) was used to delineate manual segmentation of the Renji-2021 dataset [23]. The LV endocardium and epicardium was delineated as previously described [11,18]. To obtain the right ventricular cavity, the RV wall was not included. All images were segmented by C1 and verified by another experienced cardiologist 2 (C2, 5 years of CMR experience). After manual segmentation, the Renji-2021 dataset was combined with the ACDC-2017 dataset.

#### 2.3.2. Pre-Processing

Original images and masks were processed with following steps. First, the in-plane resolutions of the images and masks were resampled to 1.0 mm × 1.0 mm using SimpleITK [24]; second, contrast-limited adaptive histogram equalization was applied to all images with the scikit-image library [25]; third, the pixels’ gray-level value underwent min–max normalization.

#### 2.3.3. Data Partition

The Private Renji-2021 dataset and ACDC-2017 dataset were combined and then randomly divided into three parts: a training dataset (60% of data, N = 350), a validation dataset (20% of data, N = 115), and an internal testing dataset (20% of data, N = 115). Within each part, patients with different types of disease were evenly distributed. The M&M-2020 dataset was independently used as an external testing dataset.

### 2.4. Machine Learning Scheme

#### 2.4.1. DL Scheme

The proposed deep learning pipeline included a localization-cropping module and a segmentation module: (a) whole heart segmentation was obtained from an UNet-like model (Localization UNet, L-Unet); (b) the center of mass (CoM) was calculated at the slice-level for each image, and the weighted CoM was defined at the subject-level according to Equations (1)–(3); (c) images were cropped around the weighted CoM and an optimal size was chosen; and (d) anatomical structure segmentation was obtained from UNet-like models (Segmentation UNets, S-Unets) with cropped images. Models were trained with the training dataset. The validation dataset was utilized to monitor the model’s performance. The internal and external testing datasets were used for the model assessment.

(1) $$X_{center}=\frac{\sum_{1}^{j}W_{j}\times X_{j}}{\sum_{1}^{j}\;W_{j}}$$

(2) $$Y_{center}=\frac{\sum_{1}^{j}W_{j}\times Y_{j}}{\sum_{1}^{j}\;W_{j}}$$

(3) $$Weighted\;Center\;of\;Mass=(X_{center},Y_{center})$$

where *W*${}_{j}$ represents the pixel number in slice *j*, *X*${}_{j}$ and *Y*${}_{j}$ represent the location of the CoM for slice *j* on the x-axis and y-axis.

#### Model Structure and Experiment Settings

**Figure 2:**
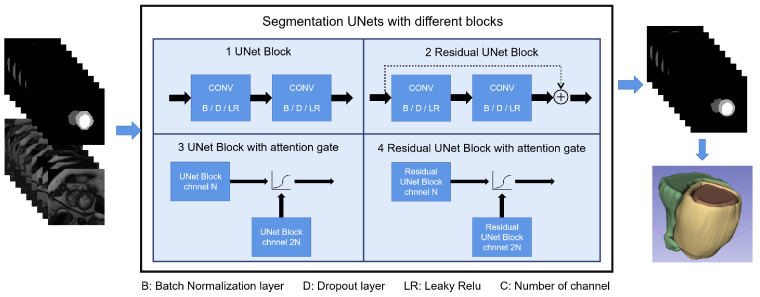
Shows the S-Unet performance with differences among the four types of disease. The four different structures of S-Unets are also provided. B/D/LR represents the batch normalization/dropout/leaky relu layer, respectively. For the 3D reconstructed images, yellow part represents the LV myocardium, the red part represents the LV cavity and the green part represents the RV cavity.

An 18-layer UNet backbone was used in both L-UNet and S-UNets but with different input shapes. A detailed model structure is shown in Figure 2. For L-UNet, after preprocessing, images maintained their height to width ratios and were resized to 256 × 256 pixels as inputs. For S-UNets, the inputs were 160 × 160 pixel precropped images, and four S-Unet model structures were included: (i) UNet; (ii) Attention UNet (A-UNet); (iii) Residual UNet (R-UNet); and (iv) Residual Attention UNet (RA-UNet).

Both L-UNet and S-UNets were trained for 200 epochs with an Adam optimizer with a 1 $\times \;10^{-3}$ initial learning rate. The optimizer scheduler monitored the validation loss and reduced the learning rate by a factor of 0.2 and a patience of 7 epochs. The minimal learning rate was set to $6.4\times 10^{-8}$; the loss functions used were DICE, cross entropy (CE), and the DICE + CE loss function (Equations (4) and (5)). Data augmentation was only used in S-UNets and included random rotation, translation, flipping, and elastic transformation using the Albumentations library [26].

(4) $$L_{DICE}=1-\frac{2\sum_{i}^{N}y_{i}p_{i}+\epsilon_{1}}{\sum_{i}^{N}y_{i}^{2}+\sum_{i}^{N}p_{i}^{2}+\epsilon_{2}}$$

(5) $$L_{CE}=-\sum_{i}^{N}y_{i}log\left(p_{i}\right)$$

*y*${}_{i}$ represents the true label of pixel *i*, while *p*${}_{i}$ represents the predicted value of pixel *i*.

#### DL Model Evaluation

The performance of the DL models was evaluated based on the dice similarity coefficient (DSC), intersection over union (IoU), precision, and recall in the testing dataset (Equations (6) and (7)).

(6) $$DSC=\frac{2|X\cap Y|}{\left|X|+|Y\right|}$$

(7) $$IoU=\frac{\left|X\cap Y\right|}{\left|X\cup Y\right|}$$

#### 2.4.2. Segmentation Quality Definition

For 2D segmentation, a DSC < 0.7 was defined as bad quality while a DSC ≥ 0.7 was defined as good quality, as in previous research [7]. For 3D segmentation, the cutoff value was set to 0.85. Therefore, for each anatomical structure, each 2D segmentation or reconstructed 3D segmentation was classified into good- or bad-quality groups according to its actual DSC value.

#### 2.4.3. Radiomics Scheme

To generate a robust radiomics dataset for further analysis, segmentations with various DSC predictions were needed. Therefore, eight weights (including the best model weight and seven suboptimal model weights) used during the model training process were selected from each S-UNet and used to generate a new segmentation dataset based on training and validation images. Therefore, we theoretically generated 32 2D/3D segmentations of varied quality at both the slice and subject levels. This dataset was used for radiomics feature extraction, feature selection, and model development. The testing data were used for the model performance evaluation, as described previously.

#### Definition of Suboptimal Models

During the segmentation model development stage, after each epoch, the validation loss was documented once validation loss decreased compared to previous epochs. The model weights were saved to the local server. After model training processes, there were many model weights saved with different validation losses. The model weight with the lowest validation loss was named the best model, while we named the other model weights suboptimal models. By using suboptimal models, we were able to generate low-quality segmentations and use these to increase our radiomics dataset and improve the model’s generalization ability.

#### Feature Extraction and Feature Selection

Radiomics features were extracted at the 2D and 3D levels from the original images. Before feature extraction, the images underwent normalization (normalization scale equals 0 to 256) and discretization (with a bin-width of 16). For 3D images, the z-axis spatial resolution was resampled to 1.0 mm in addition to achieve voxel spatial isotropy. Additionally, the radiomics features of different anatomical structures (RVC, MYO, and LVC) were extracted separately with Pyradiomics library [27]. Thereafter, we had six radiomics groups, namely RVC-2D, RVC-3D, MYO-2D, MYO-3D, LVC-2D, and LVC-3D.

After feature extraction, within each feature group, the Pearson correlation (ρ) coefficient was calculated for each feature. Features with ρ > 0.8 were defined as being highly correlated and were removed [28].

For the regression task, feature selection was performed based on the highest mutual information dependency with DSC values [29]; for the classification task, feature selection was performed based on the analysis of variance (ANOVA) F-value between the score quality (good [1] or bad [0]) and feature values. To improve the explainability of our proposed models. For model development, we considered the subject number in this study and decided to select, at most, 12 features, which was also similar to previous studies [30].

#### Regression Model Development and Evaluation

Although various DSC values were present in our radiomics dataset, to generate a balanced model, weights were calculated for good- and bad-quality groups when appropriate. Five types of regression model were tested: (1) random forest regressor (RFR), (2) gradient boost regressor (GBR), (3) K nearest neighbor regressor (KNNR), (4) linear regression regressor (LRR), and (5) multilayer perceptron regressor (MLPR). Support vector machine methods were abandoned due to their long computation times. All regressors were trained with the five-fold cross validation and grid search method to determine the best combination of parameters. The final regression model was developed with all training and validation datasets. The model performance was evaluated in the testing dataset using the mean absolute error (MAE). The prediction coefficient of determination ($R^{2}$) is also reported. The models with the best performance were selected.

#### Classification Model Development

We further developed a series of classification models based on the radiomics features to evaluate the value of the radiomics features for segmentation quality classification. The regressors’ performance was re-evaluated in the images predicted as being of good quality. Two classifiers were selected: (1) the random forest classifier (RFC) and (2) the gradient boost classifier (GBC). Since we aimed to develop a QC system, the classification performance was mainly evaluated with the negative detection rate (NDR), as shown in Equation (8).

(8) $$Negative\;Detection\;Rate\;\left(NDR\right)=\frac{TN}{FN+TN}$$

*TN* = true negative; *FN* = false negative. Negative results represent bad-quality segmentation predictions.

#### Distribution Pattern among Disease Types, Segmentation Quality, and MAE

To explore changes in the tendency of disease type/segmentation quality and MAE, density plots were plotted. Bland–Altman analyses were used to show agreement for actual DSC scores and predicted DSC scores.

### 2.5. Post Hoc Analysis

To verify the usefulness of the classification models on the QC system performance, we compared the MAE vlues of all segmentations and predicted good-quality segmentations.

With the advent of the segment anything model (SAM) [31], we chose some of our images as inputs and tested their segmentation ability with the “every” mode https://segment-anything.com/demo (accessed on 10 April 2023).

### 2.6. Statistical Analysis

SPSS (version 26) and Python (version 3.7.10) were used for the statistical analysis. The model performance was assessed using the MAE (between the predicted DSC and actual DSC) as previously described.

To compare means, student’s T tests were conducted as appropriate. Class weights were calculated when appropriate using the scikit-learn library [32].

## 3. Results

### 3.1. Study Population

As shown in Table 1, 520 subjects (N${}_{DCM}$ = 35, N${}_{HCM}$ = 227, N${}_{HHD}$ = 98, N${}_{HC}$ = 160) were obtained from Renji Hospital, 60 subjects (N${}_{DCM}$ = 20, N${}_{HCM}$ = 20, N${}_{HC}$ = 20) were obtained from the ACDC-2017 dataset, and 273 subjects (N${}_{DCM}$ = 97, N${}_{HCM}$ = 82, N${}_{HHD}$ = 15, N${}_{HC}$ = 79) were obtained from the M&M-2020 dataset. The data partition is shown in Table 2.

Results from the external testing dataset are available in the Supplementary Materials.

### 3.2. Whole Heart Localization and Cropping

Since accurate localization is important for further analysis, L-UNets with different loss functions were compared in the whole heart segmentations. As shown in Table 3, CE + DICE loss achieved the highest DSC and IoU values for whole heart area segmentation (DSC: 0.954 vs. 0.952 (CE) and 0.944 (DICE), both *p* < 0.001). Therefore, the following cropping step was based on the localization results of the L-UNet model with CE + DICE loss.

Images were cropped around the weighted CoM with different sizes (128, 160, and 192 pixels, 1.0 mm × 1.0 mm resolution per pixel). With 128 pixels, heart areas in some slices were cropped out, while with 192 pixels, the background still took up a large portion of the image area. Therefore, a final size of 160 pixels was used to crop the images.

### 3.3. Segmentation of RVC, MYO, and LVC

Figure 2 shows the segmentation pipeline and different U-net structures used in this study. The pipeline of the segmentation and reconstruction part is shown in Figure 2. The 3D segmentations were reconstructed from 2D segmentations, and the 3D DSC/IoU was calculated accordingly.

**Figure 3:**
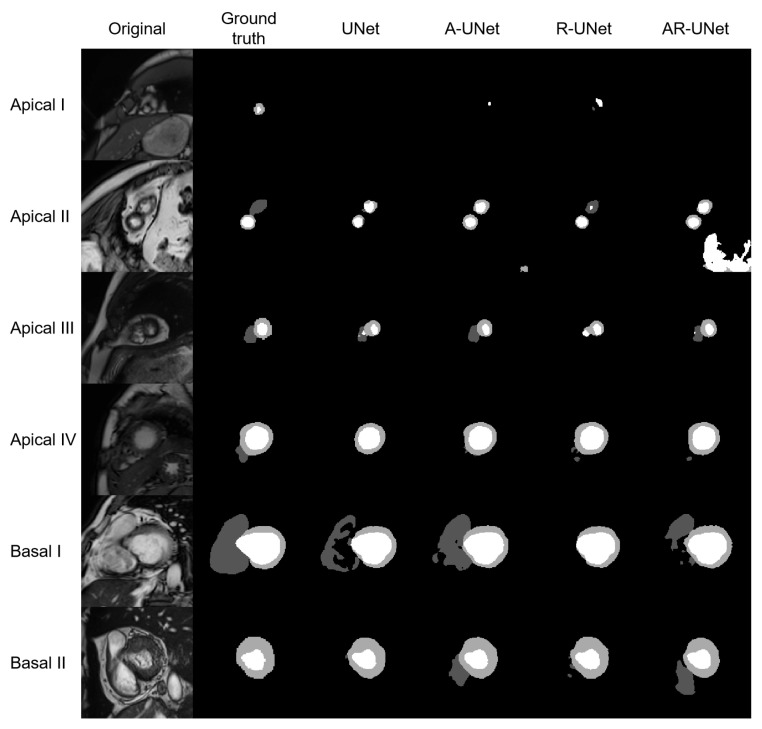
Shows six examples of low-quality segmentations, including four apical slices and two basal slices. From left to right, the original images, manually derived masks and automatic segmentations for the four S-Unets are listed, respectively. The I to IV shows that images were derived from different subjects.

The model performance of four S-UNets is shown in Table 4. From both slice-level and subject-level evaluations, the A-UNet showed the highest 2D and 3D scores within all anatomical structures (2D DSC: 0.863 for RVC, 0.872 for MYO and 0.940 for LVC; 3D DSC: 0.928 for RVC, 0.886 for MYO, and 0.962 for LVC). However, the detection of low-quality segmentations is more important in a QC system. Therefore, we visually inspected the testing segmentations and noticed that low-quality segmentations was mainly distributed at the apical or basal levels. Figure 3 shows six examples of low-quality segmentations.

### 3.4. Feature Extraction, Model Selection, and FEATURE Selection

For each S-UNet, eight models with different weights were selected and applied to the training and validation datasets, resulting in a maximum of 137,760/14,880 automatic 2D/reconstructed 3D segmentations for RVC, MYO, and LVC, respectively (the detailed segmentation numbers are shown in Supplementary Tables S1 and S2). For each 2D segmentation, 102 features were extracted, while for each 3D segmentation, 107 features were selected. The full feature lists are shown in Supplementary Tables S3 and S4.

The regression model’s performance is shown in Supplementary Table S5. After a comparison, we found that GBR showed the best performance and was selected for further analysis.

The classification model’s performance is shown in Supplementary Table S6. After a comparison, we found that RFC showed the best performance and was selected for the final classification model.

Table 5 summarizes the feature numbers used in different groups that achieved the best performance levels on the regression and classification tasks. The details of the feature name information and statistical results are provided in Supplementary Tables S7 and S8.

Table 6 shows the average actual DSC, predicted DSC, and MAE with the best-performing regression models among six groups.

### 3.5. Detailed Performance of the LVC-2D Group

**Figure 4:**
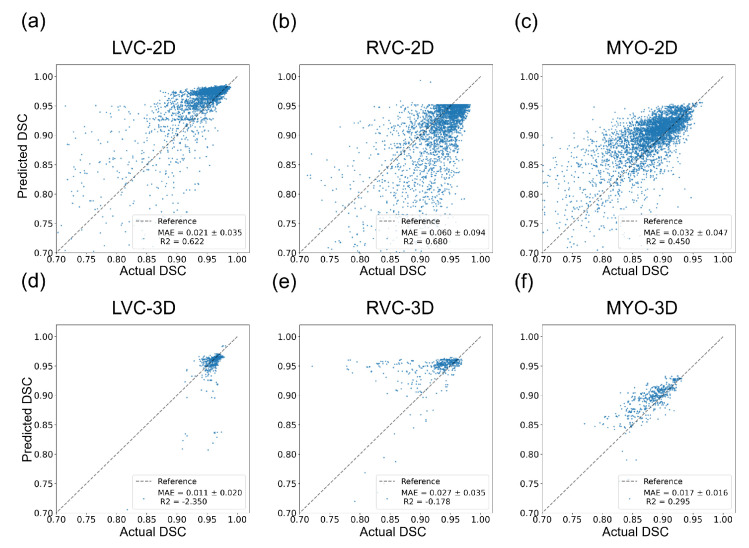
Results of regression models on the internal testing dataset for all six groups. The MAE ± SD and $R^{2}$ are shown on the figure legend. (**a**–**c**) show the results for the LVC-2D, RVC-2D, and MYO-2D groups, respectively, and (**d**–**f**) show the results for the LVC-3D, RVC-3D, and MYO-3D groups, respectively.

**Figure 5:**
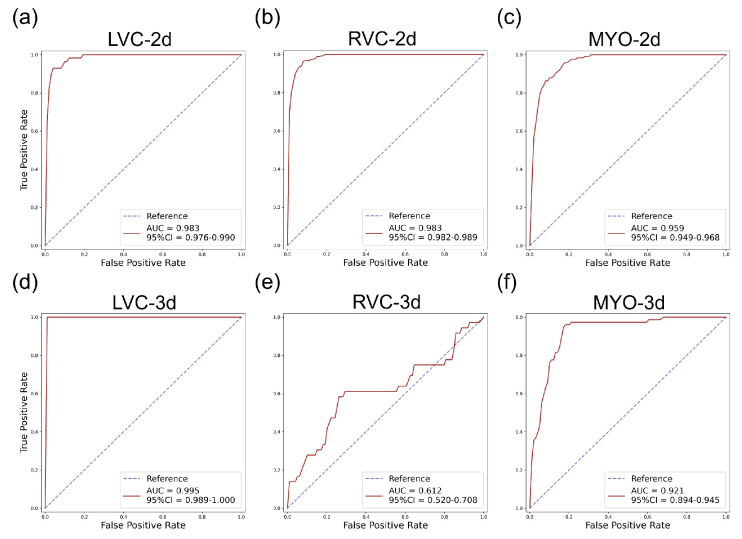
ROC curves for the internal testing data for all six subgroups. The AUC and 95% confidence interval (CI) are shown on the figure legend. Subplots (**a**–**f**) are the same as in Figure 4.

**Figure 6:**
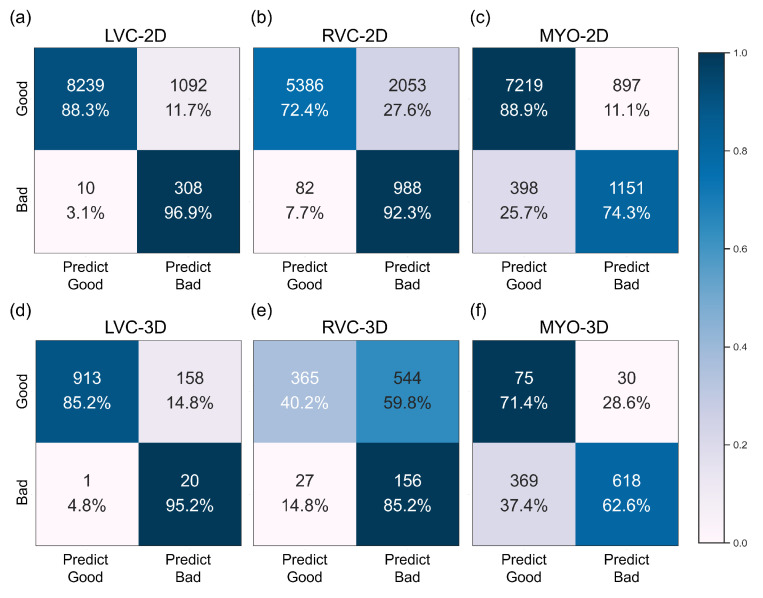
Confusion matrices of the best performance classification model for the internal dataset for all six subgroups. Subplots (**a**–**f**) are the same as in Figure 4.

**Figure 7:**
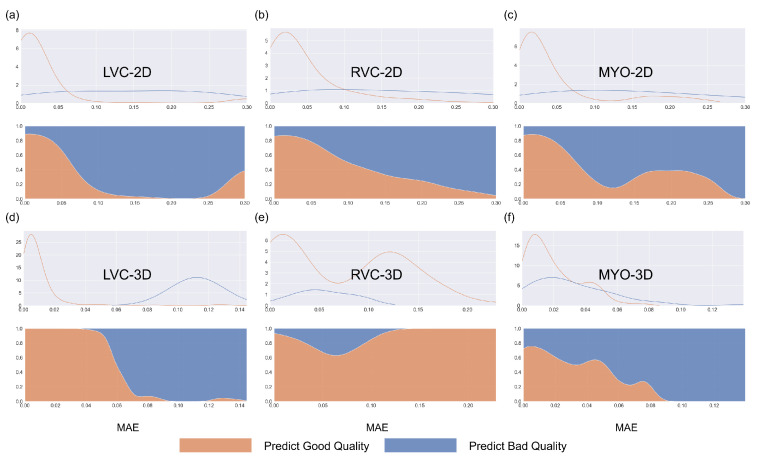
Density plots for all segmentations for the internal testing dataset for the six subgroups. Blue represents bad-quality predictions and orange represents good-quality predictions. The density plots in the first row represent changes between the number of segmentations and MAE, while the density plots in the second row (filling mode) show the relative proportions of predicted good or bad segmentations with MAE. Subplots (**a**–**f**) are the same as in Figure 4.

**Figure 8:**
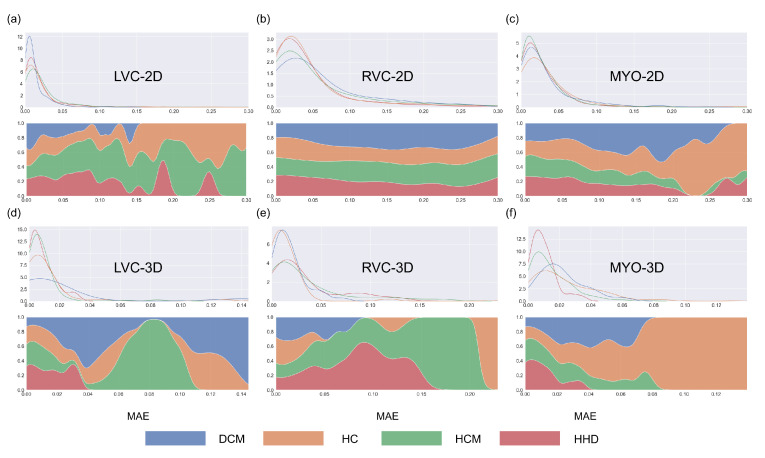
Density plots for all segmentations for the six groups. Blue, orange, green, and red represent DCM, HC, HCM, and HHD, respectively. The density plots in the first row represent changes between the number of segmentations and MAE, while the density plots in the second row (filling mode) show the relative proportions of different disease types with MAE. Subplots (**a**–**f**) are same as in Figure 4.

**Figure 9:**
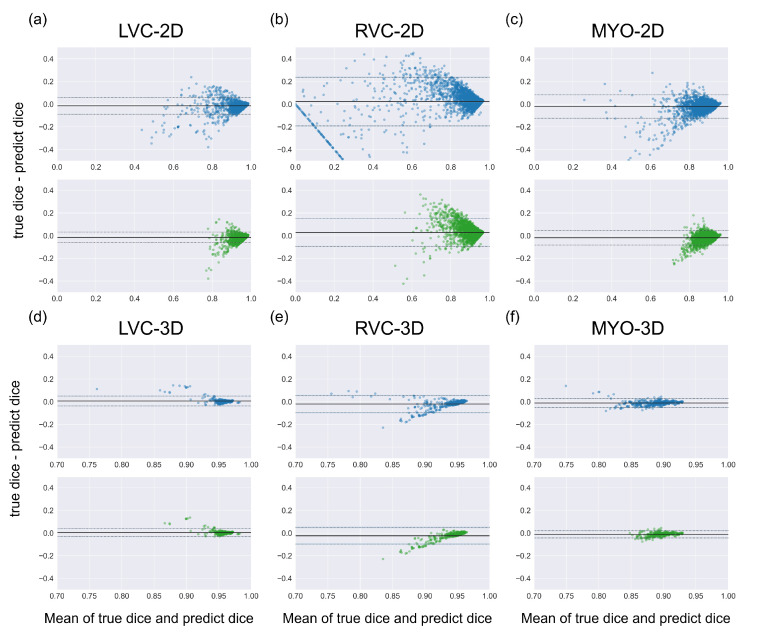
Bland Altman plots of all segmentations (the first row with blue dots) and the good-quality segmentations predicted using classification models (the second row with green dots). Comparing two Bland Altman plots, the diminished dots represented segmentations that were predicted as being of bad quality by classification models. Subplots (**a**–**f**) are the same as in Figure 4.

The results for the LVC-2D group are shown in Figure 4, Figure 5, Figure 6, Figure 7, Figure 8 and Figure 9 in subplot (a). The best regression performance was achieved with six radiomic features, and the best classification performance was also achieved with six features (the included features are available in Supplementary Tables S7 and S8. The MAE for the testing dataset was 0.021 ± 0.035 (Figure 4), the AUC was 0.983 (Figure 5), and the NDR was 93.0% (Figure 6). As Figure 7 shows, when the MAE increased, the proportion of predicted bad segmentations also increased. According to Figure 8 and Table 6, most DCM segmentations showed a MAE <of 0.15, and the *p*-value for the MAE difference between the DCM subgroup and all subjects was 0.002 (0.021 vs. 0.013). While the distribution of MAE was quite balanced among the HC, HCM, and HHD subgroups, the corresponding *p*-values were 0.560, 0.220, and 0.558, respectively. The Bland–Altman analysis (Figure 9) showed that segmentations with lower DSC values were removed with the classification model. As Table 7 shows, after the removal of predicted low-quality segmentations, the MAE improved by 0.004 compared with all other segmentation models with a *p*-value of 0.402.

### 3.6. Detailed Performance of the RVC-2D Group

The results for the RVC-2D group are shown in Figure 4, Figure 5, Figure 6, Figure 7, Figure 8 and Figure 9 in subplot (b). The best regression performance was achieved with 12 radiomic features, and the best classification performance was achieved with nine features (included features are available in Supplementary Tables S7 and S8. The MAE for the testing dataset was 0.060 ± 0.094 (Figure 4), the AUC was 0.983 (Figure 5), and the NDR was 90.0% (Figure 6). Figure 7 shows that a segmentation with a higher MAE was more likely to be detected by classification models after the exclusion of predicted low-quality segmentations. The MAE achieved an 0.019 improvement with a *p*-value of 0.106 (Table 7). As Figure 8 shows, density plots of the RVC-2D group had a balanced distribution pattern for the MAE between different pathologies. The Bland–Altman analysis (Figure 9) showed that most failed segmentations were removed by the classification model.

### 3.7. Detailed Performance of the MYO-2D Group

Results for MYO-2D group was showed in Figure 4, Figure 5, Figure 6, Figure 7, Figure 8 and Figure 9 in subplot (c), the best regression performance was achieved with 6 features and the best classification performance was achieved with 9 features (included features are available in Supplementary Tables S7 and S8. The MAE for testing dataset was 0.032 ± 0.047 (Figure 4) and the NDR was 85.5% (Figure 6). The MAE distribution patterns showed in density plots (Figure 7 and Figure 8) had similar pattern in previous 2D groups. The Bland Altman analyses (Figure 9) showed that after classification selection, low-quality segmentations were filtered. Additionally, the MAE increased by 0.006 in 2D-MYO group with *p*-value of 0.386.

### 3.8. Detailed Performance of the LVC-3D Group

The results for the LVC-3D group are shown in Figure 4, Figure 5, Figure 6, Figure 7, Figure 8 and Figure 9 in subplot (d). The best regression performance was achieved with 12 features, and the best classification performance was achieved with 12 features (included features are available in Supplementary Tables S7 and S8. The MAE for the testing dataset was 0.011 ± 0.020 (Figure 4), and the NDR was 100.0% (Figure 6). We also noticed that only one reconstructed LVC-3D segmentation was defined as being of bad quality, indicating that most reconstructed LVC-3D segmentation models showed good performance levels. The density plot presented in Figure 7 shows that almost all segmentations with MAE > 0.07 were successfully detected. The density plot presented in Figure 8 shows that the HHD subgroup had lower MAE (all <0.04), while the DCM subgroup has higher MAE, it also exhibited in Table 6. Since only one segmentation was removed, according to the segmentation model, the Bland–Altman showed minor changes, as presented in Figure 9.

### 3.9. Detailed Performance of the RVC-3D Group

The results for the RVC-3D group are shown in Figure 4, Figure 5, Figure 6, Figure 7, Figure 8 and Figure 9 in subplot (e), the best regression performance was achieved with seven features, and the best classification performance was achieved with five features (included features are available in Supplementary Tables S7 and S8. The MAE for the testing dataset was 0.027 ± 0.035; however, the $R^{2}$ was −0.178, and the NDR was 16.7%. This indicates that our classification model failed to detect low-quality segmentations in our internal testing dataset, regardless of the 3D-RVC subgroup. Therefore, only a small portion of the predicted bad quality segmentations was successfully detected, as shown in Figure 7. Meanwhile, some low-quality segmentations were wrongly predicted to be high-quality masks. As the Bland–Altman plots show, some blue dots with mean DSC < 0.5 were removed, but most outliers still remained (Figure 9). Additionally, the removed dots show a (true DSC-predict DSC) <> 0, which indicates that our classification model performed better with underestimated segmentations than overestimated segmentations.

### 3.10. Detailed Performance of the MYO-3D Group

The results for the MYO-3D group are shown in Figure 4, Figure 5, Figure 6, Figure 7, Figure 8 and Figure 9 in subplot (f). The best regression performance was achieved with 11 features, and the best classification performance was achieved with 10 features (included features are available in Supplementary Tables S7 and S8. The MAE for the testing dataset was 0.017 ± 0.016 (Figure 4), and the NDR was 75.0% (Figure 6). Most segmentations showed an MAE of <0.10, and most predicted true segmentations had an MAE of <0.08 (Figure 7); however, in Figure 8, we noticed that segmentations of MAE > 0.08 belong to the HC subgroup. This is also reflected in Table 6 by the *p*-value of the HC subgroup < 0.001. The Bland–Altman analysis showed great results for the predicted good-quality segmentations. Almost all segmentations were located in the mean ± 1.96 SD area (Figure 9).

### 3.11. Differences between 2D and 3D Groups

As shown in Figure 4, Figure 5, Figure 6, Figure 7, Figure 8 and Figure 9, first of all, the 2D groups had more samples than the 3D groups for the training, validation, and testing datasets. As the scatter plots in Figure 4 show, the 2D groups showed higher $R^{2}$ values than the 3D groups (2D groups: 0.622, 0.680, and 0.450 vs. 3D groups: −2.350, −0.178, and 0.295), which indicates that the 2D groups showed more robust radiomics-based DSC prediction models than the 3D groups. For the confusion matrices and ROC curve analysis, except for the LVC-3D group, the 2D groups also showed better AUC values and higher NDR values. As for the density plots (Figure 8) between MAE and the disease types, the distribution of 2D MAE was more balanced compared with 3D groups. This phenomenon was also confirmed by the results presented in Table 6.

### 3.12. Post Hoc Analysis

**Figure 10:**
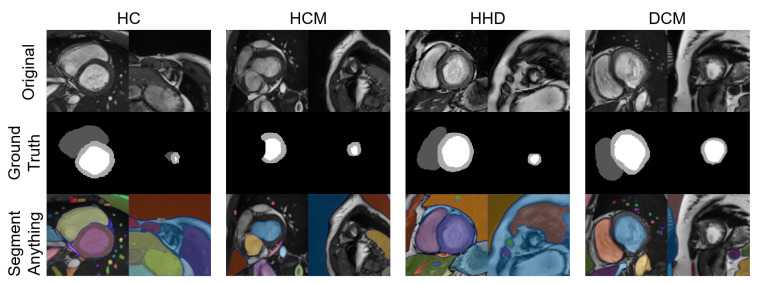
Eight images with manual segmentations and SAM segmentations. For each disease type, we selected an apical slice and a basal slice.

A total of eight images were tested with the SAM “every” mode. The segmentation results are shown together with the manual segmentations in Figure 10. The results of the SAM showed an acceptable performance on the basal slices; however, for the apical slices, none of the four apical slices showed acceptable results.

### 3.13. Results for the External Dataset

The models’ performance levels on the external testing dataset are available in Supplementary Figures S1–S6 and Supplementary Tables S9–S13.

## 4. Discussion

In this study, we developed an analysis platform that incorporates a DL-based automatic segmentation cine and a radiomics-based QC for short-axis CMR cine. To achieve this, we first developed a localization and segmentation pipeline using U-net models. Thereafter, we developed a two-stage radiomics-based quality control (QC) system for automatic segmentations. Our hypothesis, that radiomics features could facilitate the QC of automatic segmentations, was validated through experiments. By using RF classifiers and GB regressors, our methods exhibited a high mal-segmentation detection rate and accurate DSC estimation in most situations.

### 4.1. Discussion Regarding Model Performance

#### 4.1.1. DL Model Performance

As shown in Table 4, our DSC scores for 2D segmentations were 0.863, 0.940, and 0.872 for RVC, LVC, and MYO, respectively. These results demonstrate that our model structure is suitable for the segmentation task. However, as depicted in Figure 3, our four S-Unets exhibited a decreased segmentation performance on certain apical and basal slices. This phenomenon was also observed in the recent SAM segmentations (Figure 10). During our analysis, we observed that S-Unets with various modifications (residual, attention parts) exhibited slightly varying performances on ambiguous regions of interest (ROIs). For instance, S-Unets equipped with attention structures tended to segment the right ventricular cavity more accurately in basal slices, while those with residual parts displayed better performance levels on smaller ROIs (Figure 3). Moreover, the variability of low-quality segmentations offered unique samples for our radiomics dataset. This variability partially explains why we incorporated modified model structures in our analysis pipeline.

#### 4.1.2. QC Performance on Our Dataset

The regression models performed well on all subgroups in the training dataset with better performances seen in the 2D subgroups, as characterized by the $R^{2}$ values (Figure 4). However, this difference could be partially attributed to the larger sample size used in the 2D segmentations. We further utilized RF classifiers and found that, in the 2D subgroups, all NDRs were above 85% (Figure 6), indicating that the radiomic-feature-based classification models could effectively identify low-quality segmentations.

One notable exception was the 3D-RVC group, which exhibited an NDR of only 16.7%. Upon examining the density plots for the MAE (Figure 7), we observed that as the MAE increased, the proportion of predicted bad-quality segmentations also increased for all segmentation results (with the exception of the 3D-RVC group). The distribution pattern of 2D MAE was relatively balanced across the various disease groups (Figure 8). With the failure of our model in the RVC-3D subgroup, we checked the radiomics features included in Supplementary Table S5, and we found that most include features belonging to the shape feature family. Due to the underlying pathology, the shape variance of the RVC is much higher than those of LVC or MYO (the RVC shape is more sensitive to external changes, such as myocardium hypertrophy or hemodynamic changes). Additionally, the segmentation models failed to segment many RVC apical slices. After reconstruction, this could lead to the instability of 3D-RVC features.

By comparing two Bland–Altman plots for each subgroup, we demonstrated the prediction results for all segmentations and predicted good-quality segmentations. We also noticed that most excluded segmentation instances were located outside of the >1.96 SD interval, as shown in Figure 9. We also noticed a group of awkward points in the Bland–Altman plots, especially in the RVC-2D subgroup. For an image with an actual DSC equal to 0 and a predicted dice equal to D${}_{i}$ (means the segmentation model segmented some irrelevant area as a ROI), the *x* axis location for that point is 0.5D${}_{i}$ and the *y* axis location is −D${}_{i}$. This could explain why all of those awkward points are located on the line ‘y = −2x’. We also examined our segmentation performance retrospectively and found that apical slices were hard to segment in some subjects. By using Bland–Altman plots, those low-quality segmentations were obvious in our 2D-RVC group. Luckily, our classification model successfully detected most of these segmentations.

### 4.2. QC Performance Compared with Previous Methods

We compared our methods with a previous RCA method [7] and a DL-based method [8]. In Robinson’s work, they performed experiments on several datasets with 2D segmentations for the RVC, LVC, and MYO subgroups. The MAE values were 0.030–0.146, 0.020–0.082, and 0.044–0.268, respectively, compared to our results where the MAE values for RVC, LVC, and MYO were 0.060, 0.021, and 0.032. One obvious drawback of the RCA method is the need for a reference dataset. A larger reference dataset could lead to overestimation of the segmentations’ quality. For the 3D segmentation QC performance, we compared our results with previous DL-based QC results. The DSC for MYO was 0.017 ± 0.016 (ours) vs. 0.016 ± 0.028 (DL); for LVC, it was 0.011 ± 0.020 (ours) vs. 0.012 ± 0.017 (DL). These comparison results show that our models perform well for automatic QC regarding the LVC and MYO structures. However, experiments were not performed on the RVC subgroup.

### 4.3. QC with the Mature Segmentation Model

In contrast to diagnostic applications, a QC system should prioritize the detection of bad-quality segmentations, rather than improving the classification accuracy. As a result, we chose the NDR as our primary criterion for the classification evaluation. Additionally, we observed that deep learning segmentation models for cardiac segmentation are currently well-developed. The reported average DSC is 0.85–0.97 for the ACDC-2017 dataset and M&M challenge dataset with various DL model structures [3,33,34,35]. As previously mentioned, mal-segmentations at the slice level are primarily distributed in the apical or basal regions of the heart. However, these low-quality segmentations have little effect on the 3D DSC prediction. This partially explains why the 3D DSC is higher than the 2D DSC (refer to Table 4). However, it is important to note that the absence of apical or basal slice segmentations can significantly impact the 3D radiomics features, such as the maximal long axis length, particularly in the shape feature group. This phenomenon was also observed in our experiment (Supplementary Tables S7 and S8). Meanwhile, although coarse borders may not significantly impact the 2D or 3D DSC, certain radiomics features are sensitive to edges, as demonstrated in previous studies [36,37,38]. The characteristics exhibited by radiomics-based quality control systems provide a new evaluation perspective compared to previous methods. This makes radiomics an ideal method for automatic segmentation evaluation and quality control. As shown in Figure 10, the segmentation of apical slices remains challenging, which is why a quality control system is necessary, even with the use of large models, such as SAM.

### 4.4. Technical Innovations and Clinical Insights

To the best of our knowledge, this is the first study to utilize radiomics as a quality control tool for automatic cardiac magnetic resonance (CMR) segmentations. Our findings demonstrate that radiomics techniques yield great DSC prediction results. In addition, our method is capable of efficiently detecting mal-segmentations with NDR values greater than 0.85 in all 2D groups, achieving values of 90.0% [RVC], 93.0% [LVC], and 85.5% [MYO]. As a previous comparison showed, our models showed great QC performances for both 2D and 3D segmentations.

Our method is also computationally friendly. In our case, with a NVIDIA RTX 3090 GPU and an AMD 3900X CPU, the training time for the localization model and segmentation model was less than 5 h. We also tested our training model with a NVIDIA RTX 3060 (12 GB memory), which is also capable of carrying out the training process. More importantly, the Unets used in this study were examined in various tasks with numerous variants, and every center was able to develop dedicated models.

In this study, we aimed to provide a new perspective for QC, and we tested the feasibility of our proposed method. Once the quality control pipeline is built, the operator only needs to decide the ED frame, and the computation time is <10 s for each instance, which is 60 times faster than that of the RCA method, as previously reported [7]. With a short computation time, our method has the application potential for real-time DSC predictions in a clinical scenario. Timely detection of low-quality segmentations could save time for researchers and reduce the human workload.

This method has another obvious advantage compared with the RCA method: we do not need to select a reference dataset as in the RCA method [7]. Additionally, we do not need to test the reproducibility of selected features. The manually derived ROIs were only used for calculating the DSCs of automatic segmentations. The radiomics features of manual segmentations were not extracted or analyzed.

### 4.5. Limitations

This study had several limitations. Firstly, while we did include the ACDC-2017 and M&M-2020 datasets, the majority of data for our training and validation datasets (nearly 90%) were derived from a single center (Renji Hospital). Secondly, from a practical perspective, most dedicated segmentation models have shown great results. To address this, we included suboptimal models for the radiomics training dataset generation. However, during the testing phase, we only used segmentations from the optimal S-UNets to evaluate the model performance for both regression and classification. Thirdly, radiomic features can only be extracted from images that have specified ROIs. Therefore, our method is not applicable to images that lack segmentations. However, the missing information may be reflected in the 3D radiomics characteristics. Fourth, the contours of different structures are more clear in the ED phase than in the ES phase; therefore, only the ED phase was selected in this study.

## 5. Conclusions

In our proposed deep radiomics-based segmentation and quality control system, subjects with different disease types are analyzed, and the segmentation quality is evaluated at both the 2D and 3D levels. Our results prove that this deep radiomics approach can successfully identify “poor quality” segmentations with a high NDR and achieve a low MAE among all anatomical groups.

## Figures and Tables

**Table 1:** Shows the numbers of subjects across different diseases and datasets.

Disease Types	Training, Validation, and Internal Testing		External Testing
	Renji-2021 (N = 520)	ACDC-2017 (N = 60)	M&M-2020 (N = 273)
HCM	227	20	82
HHD	98	0	15
DCM	35	20	97
HC	160	20	79

**Table 2:** Shows the compositions of the training, validation, and testing datasets.

Disease Types	Training (N = 350)	Validation (N = 115)	Internal Testing (N = 115)	External Testing (N = 273)
HCM	10 + 139 ^1^	4 + 45 ^1^	6 + 43 ^1^	82
HHD	0 + 60	0 + 19	0 + 19	15
DCM	13 + 20	2 + 9	5 + 6	97
HC	12 + 96	2 + 34	6 + 30	79

^1^ In these columns, the first number indicates the number of subjects from the ACDC dataset, while the second number indicates the number of subjects from the Renji dataset.

**Table 3:** Shows the localization model’s performance in the internal testing dataset with different loss functions.

Loss Function	DSC	IoU
CE	0.944 ± 0.084	0.901 ± 0.104
DICE	0.952 ± 0.060	0.912 ± 0.086
CE + DICE	0.954 ± 0.056	0.916 ± 0.081

CE: cross entropy, DSC: dice similarity coefficient, IoU: intersection over union.

**Table 4:** DSC and IoU for 2D and 3D anatomical structure segmentations with four S-UNets in the internal testing dataset.

Anatomical	Parameter		2D			3D	
Structure			DSC	IoU		DSC	IoU
RVC	Unet		0.854 ± 0.236	0.794 ± 0.238		0.927 ± 0.042	0.866 ± 0.067
	R-Unet		0.853 ± 0.233	0.790 ± 0.235		0.921 ± 0.041	0.856 ± 0.067
	A-Unet		0.863 ± 0.229	0.804 ± 0.230		0.928 ± 0.040	0.869 ± 0.066
	RA-Unet		0.858 ± 0.229	0.796 ± 0.230		0.923 ± 0.039	0.860 ± 0.065
MYO	Unet		0.871 ± 0.085	0.779 ± 0.104		0.885 ± 0.026	0.794 ± 0.042
	R-Unet		0.860 ± 0.098	0.763 ± 0.113		0.877 ± 0.028	0.782 ± 0.044
	A-Unet		0.872 ± 0.091	0.781 ± 0.106		0.886 ± 0.028	0.796 ± 0.045
	RA-Unet		0.862 ± 0.101	0.768 ± 0.115		0.879 ± 0.030	0.785 ± 0.047
LVC	Unet		0.939 ± 0.088	0.895 ± 0.107		0.960 ± 0.011	0.923 ± 0.020
	R-Unet		0.936 ± 0.097	0.890 ± 0.114		0.959 ± 0.010	0.922 ± 0.018
	A-Unet		0.940 ± 0.099	0.897 ± 0.113		0.962 ± 0.011	0.926 ± 0.020
	RA-Unet		0.938 ± 0.091	0.893 ± 0.111		0.958 ± 0.017	0.920 ± 0.029

Values are means ± standard deviations (SDs). A-Unet: Attention Unet, R-Unet: Residual Unet, RA-Unet: Residual Attention Unet.

**Table 5:** Summarizes the feature numbers selected for the best performance regression and classification models.

	Regression	Classification
RVC-2D	12	9
MYO-2D	6	9
LVC-2D	6	6
RVC-3D	7	5
MYO-3D	11	10
LVC-3D	12	12

The maximal feature number was set to 12. For different tasks and cardiac structures, the feature number varied.

**Table 6:** Shows the actual DSC, predicted DSC, and MAE for different anatomical structure segmentations among different types of disease.

	Parameters	ALL	DCM	HC	HCM	HHD	p-DCM ^1^	p-HC ^2^	p-HCM ^3^	p-HHD ^4^
RVC-2D	Actual DSC	0.874	0.880	0.907	0.845	0.883				
	Pred DSC	0.852	0.850	0.876	0.829	0.865				
	MAE	0.060	0.068	0.054	0.068	0.048	0.483	0.464	0.345	0.259
LVC-2D	Actual DSC	0.943	0.958	0.949	0.934	0.946				
	Pred DSC	0.957	0.968	0.963	0.950	0.961				
	MAE	0.021	0.013	0.018	0.025	0.018	**0.002**	0.560	0.220	0.558
MYO-2D	Actual DSC	0.870	0.863	0.841	0.886	0.881				
	Pred DSC	0.890	0.888	0.868	0.903	0.898				
	MAE	0.032	0.033	0.040	0.028	0.028	0.815	0.232	0.363	0.441
RVC-3D	Actual DSC	0.925	0.935	0.944	0.911	0.918				
	Pred DSC	0.946	0.931	0.955	0.941	0.947				
	MAE	0.027	0.018	0.015	0.035	0.033	0.096	**<0.001**	**0.010**	0.133
LVC-3D	Actual DSC	0.960	0.965	0.964	0.955	0.959				
	Pred DSC	0.953	0.951	0.955	0.951	0.957				
	MAE	0.011	0.019	0.012	0.009	0.009	**0.017**	0.698	0.314	0.344
MYO-3D	Actual DSC	0.882	0.872	0.860	0.895	0.894				
	Pred DSC	0.893	0.881	0.873	0.908	0.901				
	MAE	0.017	0.022	0.024	0.015	0.010	0.100	**<0.001**	**0.046**	**<0.001**

^1–4^ *p*-values were calculated between the DCM [1], HC [2], HCM [3], and HHD [4] subgroups and the ALL group, respectively.

**Table 7:** MAE improvement among different anatomical structures for all segmentations and predicted good-quality segmentations.

	Parameter	Anatomical Structure		
		RVC	MYO	LVC
2D	MAE improvement	0.019	0.006	0.004
	*p* value	0.106	0.386	0.402
3D	MAE improvement	0.001	0.002	0.001
	*p* value	0.815	0.042	0.296

## Data Availability

No new data were created. We apologize, but the Renji-2021 dataset is unavailable due to privacy and ethical restrictions. The ACDC-2017 dataset is available at https://www.creatis.insa-lyon.fr/Challenge/acdc/databases.html upon request.

## Supplementary Materials

The following supporting information can be downloaded at: https://www.mdpi.com/article/10.3390/bioengineering10070791/s1, Figure S1: Results of regression models on external testing data among all six groups; Figure S2: ROC curves on external testing data among all six groups; Figure S3: Confusion matrices of best performance classification model among all six groups on external testing dataset; Figure S4: Density plots for all segmentations among six groups and classification results on external testing dataset; Figure S5: Density plots for all segmentations among six groups within different diseases on external testing dataset; Figure S6: Bland Altman plots of all segmentations and predicted good quality segmentations using classification models in external testing dataset; Table S1: Shows the number of 2D and 3D segmentations for radiomics training dataset; Table S2: Shows the number of 2D and 3D segmentations for radiomics testing dataset; Table S3: Shows the full feature name list extracted in 3D images; Table S4: Shows the full feature name list extracted in 2D images; Table S5: Shows the best DSC prediction MAE of different structures with five regression models in internal testing dataset; Table S6: Shows the best classification model performance of different structures with RF classifiers in internal testing dataset; Table S7: Shows the top 12 features with the highest mutual information score among 6 groups; Table S8: Shows the top 12 features with the highest highest F-statistics among 6 groups; Table S9: Localization (whole heart segmentation) performance on independent external testing dataset; Table S10: RVC, LVC and MYO segmentation performance on independent external testing dataset; Table S11: Classification model performance on independent external testing dataset with RF classifier; Table S12: Regression model performance on independent external testing dataset with GB regressor; Table S13: MAE improvement among different anatomical structures for all segmentations and predicted good quality segmentations.

## Abbreviations

The following abbreviations are used frequently in this manuscript:

AUC	area under the curve
CMR	cardiovascular magnetic resonance
DL	deep learning
DSC	dice similarity coefficient
HC	healthy control
HCM	hypertrophic cardiomyopathy
HHD	hypertensive heart disease
LVC	left ventricular cavity
MYO	myocardium
RCA	reverse classification accuracy
ROC	receiver operating curve
RVC	right ventricular cavity

## References

[1] Ibanez B., Aletras A.H., Arai A.E., Arheden H., Bax J., Berry C., Bucciarelli-Ducci C., Croisille P., Dall’Armellina E., Dharmakumar R. (2019). Cardiac MRI Endpoints in Myocardial Infarction Experimental and Clinical Trials. J. Am. Coll. Cardiol..

[2] Ommen S.R., Mital S., Burke M.A., Day S.M., Deswal A., Elliott P., Evanovich L.L., Hung J., Joglar J.A., Writing Committee Members (2020). 2020 AHA/ACC Guideline for the Diagnosis and Treatment of Patients with Hypertrophic Cardiomyopathy: Executive Summary: A Report of the American College of Cardiology/American Heart Association Joint Committee on Clinical Practice Guidelines. J. Am. Coll. Cardiol..

[3] Campello V.M., Gkontra P., Izquierdo C., Martin-Isla C., Sojoudi A., Full P.M., Maier-Hein K., Zhang Y., He Z., Ma J. (2021). Multi-Centre, Multi-Vendor and Multi-Disease Cardiac Segmentation: The M&Ms Challenge. IEEE Trans. Med. Imaging.

[4] Kohlberger T., Singh V., Alvino C., Bahlmann C., Grady L., Ayache N., Delingette H., Golland P., Mori K. (2012). Evaluating Segmentation Error without Ground Truth. Proceedings of the Medical Image Computing and Computer-Assisted Intervention—MICCAI 2012.

[5] Albà X., Lekadir K., Pereañez M., Medrano-Gracia P., Young A.A., Frangi A.F. (2018). Automatic initialization and quality control of large-scale cardiac MRI segmentations. Med. Image Anal..

[6] Valindria V.V., Lavdas I., Bai W., Kamnitsas K., Aboagye E.O., Rockall A.G., Rueckert D., Glocker B. (2017). Reverse Classification Accuracy: Predicting Segmentation Performance in the Absence of Ground Truth. IEEE Trans. Med. Imaging.

[7] Robinson R., Valindria V.V., Bai W., Oktay O., Kainz B., Suzuki H., Sanghvi M.M., Aung N., Paiva J.M., Zemrak F. (2019). Automated quality control in image segmentation: Application to the UK Biobank cardiovascular magnetic resonance imaging study. J. Cardiovasc. Magn. Reson..

[8] Fournel J., Bartoli A., Bendahan D., Guye M., Bernard M., Rauseo E., Khanji M.Y., Petersen S.E., Jacquier A., Ghattas B. (2021). Medical image segmentation automatic quality control: A multi-dimensional approach. Med. Image Anal..

[9] Li K., Yu L., Heng P.A. (2022). Towards reliable cardiac image segmentation: Assessing image-level and pixel-level segmentation quality via self-reflective references. Med. Image Anal..

[10] Mancio J., Pashakhanloo F., El-Rewaidy H., Jang J., Joshi G., Csecs I., Ngo L., Rowin E., Manning W., Maron M. (2022). Machine learning phenotyping of scarred myocardium from cine in hypertrophic cardiomyopathy. Eur. Heart J. Cardiovasc. Imaging.

[11] Neisius U., El-Rewaidy H., Nakamori S., Rodriguez J., Manning W.J., Nezafat R. (2019). Radiomic Analysis of Myocardial Native T1 Imaging Discriminates between Hypertensive Heart Disease and Hypertrophic Cardiomyopathy. JACC Cardiovasc. Imaging.

[12] Wang J., Li Y., Yang F., Bravo L., Wan K., Xu Y., Cheng W., Sun J., Zhu Y., Zhu T. (2020). Fractal Analysis: Prognostic Value of Left Ventricular Trabecular Complexity Cardiovascular MRI in Participants with Hypertrophic Cardiomyopathy. Radiology.

[13] Maffei N., Manco L., Aluisio G., D’Angelo E., Ferrazza P., Vanoni V., Meduri B., Lohr F., Guidi G. (2021). Radiomics classifier to quantify automatic segmentation quality of cardiac sub-structures for radiotherapy treatment planning. Phys. Med..

[14] Sunoqrot M.R.S., Selnæs K.M., Sandsmark E., Nketiah G.A., Zavala-Romero O., Stoyanova R., Bathen T.F., Elschot M. (2020). A Quality Control System for Automated Prostate Segmentation on T2-Weighted MRI. Diagnostics.

[15] Sakai M., Nakano H., Kawahara D., Tanabe S., Takizawa T., Narita A., Yamada T., Sakai H., Ueda M., Sasamoto R. (2020). Detecting MLC modeling errors using radiomics-based machine learning in patient-specific QA with an EPID for intensity-modulated radiation therapy. Med. Phys..

[16] Wootton L.S., Nyflot M.J., Chaovalitwongse W.A., Ford E. (2018). Error Detection in Intensity-Modulated Radiation Therapy Quality Assurance Using Radiomic Analysis of Gamma Distributions. Int. J. Radiat. Oncol. Biol. Phys..

[17] Branco L.R.F., Ger R.B., Mackin D.S., Zhou S., Court L.E., Layman R.R. (2019). Technical Note: Proof of concept for radiomics-based quality assurance for computed tomography. J. Appl. Clin. Medical Phys..

[18] Bernard O., Lalande A., Zotti C., Cervenansky F., Yang X., Heng P.A., Cetin I., Lekadir K., Camara O., Gonzalez Ballester M.A. (2018). Deep Learning Techniques for Automatic MRI Cardiac Multi-Structures Segmentation and Diagnosis: Is the Problem Solved?. IEEE Trans. Med. Imaging.

[19] UK, Constantinos O’Mahony (2014). 2014 ESC Guidelines on diagnosis and management of hypertrophic cardiomyopathy: The Task Force for the Diagnosis and Management of Hypertrophic Cardiomyopathy of the European Society of Cardiology (ESC). Eur. Heart J..

[20] Lang R.M., Badano L.P., Mor-Avi V., Afilalo J., Armstrong A., Ernande L., Flachskampf F.A., Foster E., Goldstein S.A., Kuznetsova T. (2014). Recommendations for Cardiac Chamber Quantification by Echocardiography in Adults: An Update from the American Society of Echocardiography and the European Association of Cardiovascular Imaging. J. Am. Soc. Echocardiogr..

[21] Narkiewicz K., Redon J. (2013). 2013 ESH/ESC Guidelines for the Management of Arterial Hypertension. Eur. Heart J..

[22] Merlo M., Daneluzzi C., Mestroni L., Cannatà A., Sinagra G., Sinagra G., Merlo M., Pinamonti B. (2019). Historical Terminology, Classifications, and Present Definition of DCM. Dilated Cardiomyopathy.

[23] Yushkevich P.A., Piven J., Hazlett H.C., Smith R.G., Ho S., Gee J.C., Gerig G. (2006). User-guided 3D active contour segmentation of anatomical structures: Significantly improved efficiency and reliability. NeuroImage.

[24] Lowekamp B.C., Chen D.T., Ibáñez L., Blezek D. (2013). The Design of SimpleITK. Front. Neuroinform..

[25] van der Walt S., Schönberger J.L., Nunez-Iglesias J., Boulogne F., Warner J.D., Yager N., Gouillart E., Yu T. (2014). scikit-image: Image processing in Python. PeerJ.

[26] Buslaev A., Iglovikov V.I., Khvedchenya E., Parinov A., Druzhinin M., Kalinin A.A. (2020). Albumentations: Fast and Flexible Image Augmentations. Information.

[27] van Griethuysen J.J., Fedorov A., Parmar C., Hosny A., Aucoin N., Narayan V., Beets-Tan R.G., Fillion-Robin J.C., Pieper S., Aerts H.J. (2017). Computational Radiomics System to Decode the Radiographic Phenotype. Cancer Res..

[28] Liu Q., Lu Q., Chai Y., Tao Z., Wu Q., Jiang M., Pu J. (2023). Papillary Muscle Derived Radiomic Features for Hypertrophic Cardiomyopathy Versus Hypertensive Heart Disease Classification. Diagnostics.

[29] Sulaiman M.A., Labadin J. Feature selection based on mutual information. Proceedings of the 2015 9th International Conference on IT in Asia (CITA).

[30] Zhou K., Shang J., Guo Y., Ma S., Lv B., Zhao N., Liu H., Zhang J., Xv L., Wang Y. (2023). Incremental Diagnostic Value of Radiomics Signature of Pericoronary Adipose Tissue for Detecting Functional Myocardial Ischemia: A Multicenter Study. Eur. Radiol..

[31] Kirillov A., Mintun E., Ravi N., Mao H., Rolland C., Gustafson L., Xiao T., Whitehead S., Berg A.C., Lo W.Y. (2023). Segment Anything. arXiv.

[32] Pedregosa F., Varoquaux G., Gramfort A., Michel V., Thirion B., Grisel O., Blondel M., Prettenhofer P., Weiss R., Dubourg V. (2011). Scikit-learn: Machine Learning in Python. J. Mach. Learn. Res..

[33] Zotti C., Luo Z., Lalande A., Jodoin P.M. (2019). Convolutional Neural Network with Shape Prior Applied to Cardiac MRI Segmentation. IEEE J. Biomed. Health Inform..

[34] Isensee F., Jaeger P.F., Full P.M., Wolf I., Engelhardt S., Maier-Hein K.H., Pop M., Sermesant M., Jodoin P.M., Lalande A., Zhuang X., Yang G., Young A., Bernard O. (2018). Automatic Cardiac Disease Assessment on cine-MRI via Time-Series Segmentation and Domain Specific Features. Statistical Atlases and Computational Models of the Heart. ACDC and MMWHS Challenges.

[35] Simantiris G., Tziritas G. (2020). Cardiac MRI Segmentation with a Dilated CNN Incorporating Domain-Specific Constraints. IEEE J. Sel. Top. Signal. Process..

[36] Piantadosi G., Sansone M., Fusco R., Sansone C. (2020). Multi-planar 3D breast segmentation in MRI via deep convolutional neural networks. Artif. Intell. Med..

[37] Alilou M., Prasanna P., Bera K., Gupta A., Rajiah P., Yang M., Jacono F., Velcheti V., Gilkeson R., Linden P. (2021). A Novel Nodule Edge Sharpness Radiomic Biomarker Improves Performance of Lung-RADS for Distinguishing Adenocarcinomas from Granulomas on Non-Contrast CT Scans. Cancers.

[38] Bhatia A., Birger M., Veeraraghavan H., Um H., Tixier F., McKenney A.S., Cugliari M., Caviasco A., Bialczak A., Malani R. (2019). MRI radiomic features are associated with survival in melanoma brain metastases treated with immune checkpoint inhibitors. Neuro-Oncology.

