# Supplementary material for: Radiomics-Based Quality Control System for Automatic Cardiac Segmentation: A Feasibility Study
Source: Bioengineering (Basel). 2023 Jul 1;10(7):791. doi: 10.3390/bioengineering10070791 (PMC10376472; doi:10.3390/bioengineering10070791)
Supplement: Supplementary file 1 [file bioengineering-10-00791-s001.zip › bioengineering-2368654-supplementary.pdf]

Radiomics based quality control system for automatic cardiac segmentation: a feasibility study

Supplementary Materials

Supplementary figures Pages 2-7

Supplementary tables Pages 8-23

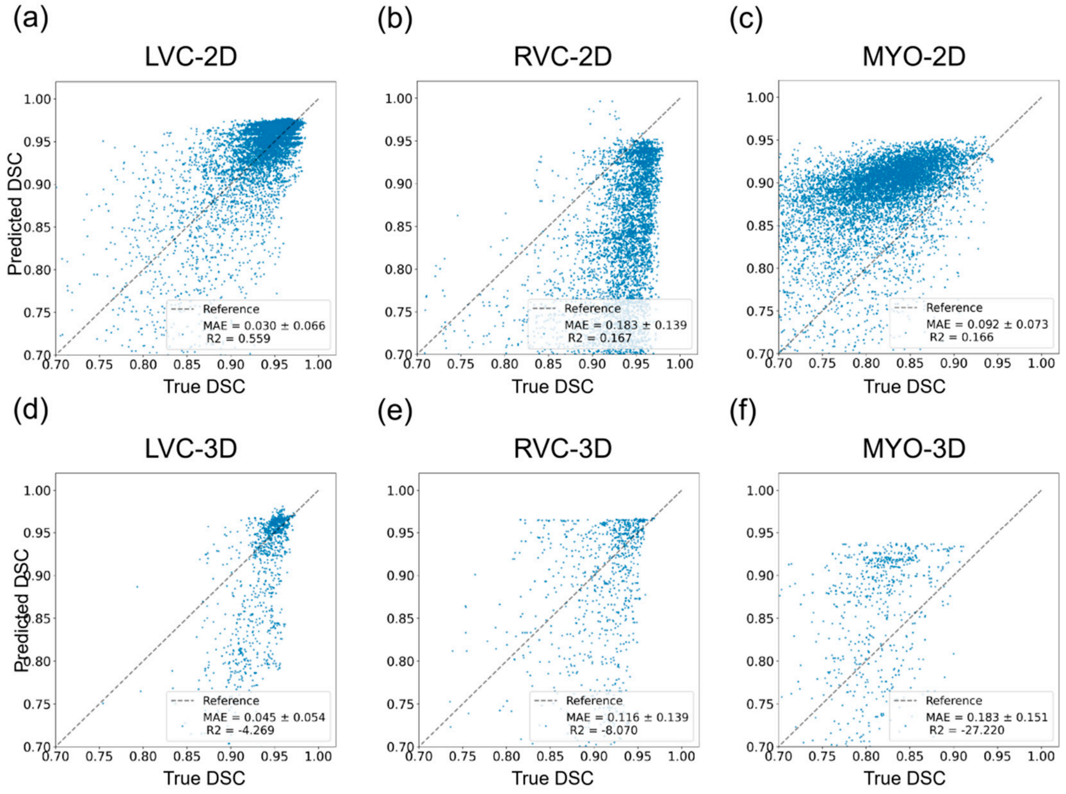

Supplementary Figure S1. Results of regression models on external testing data among all six groups, the MAE ± SD and R2 was showed on the figure legend. (a)-(c) show results on LVC-2D, RVC-2D and MYO-2D groups, respectively and (d)-(f) show results of LVC-3D, RVC-3D and MYO-3D groups, respectively.

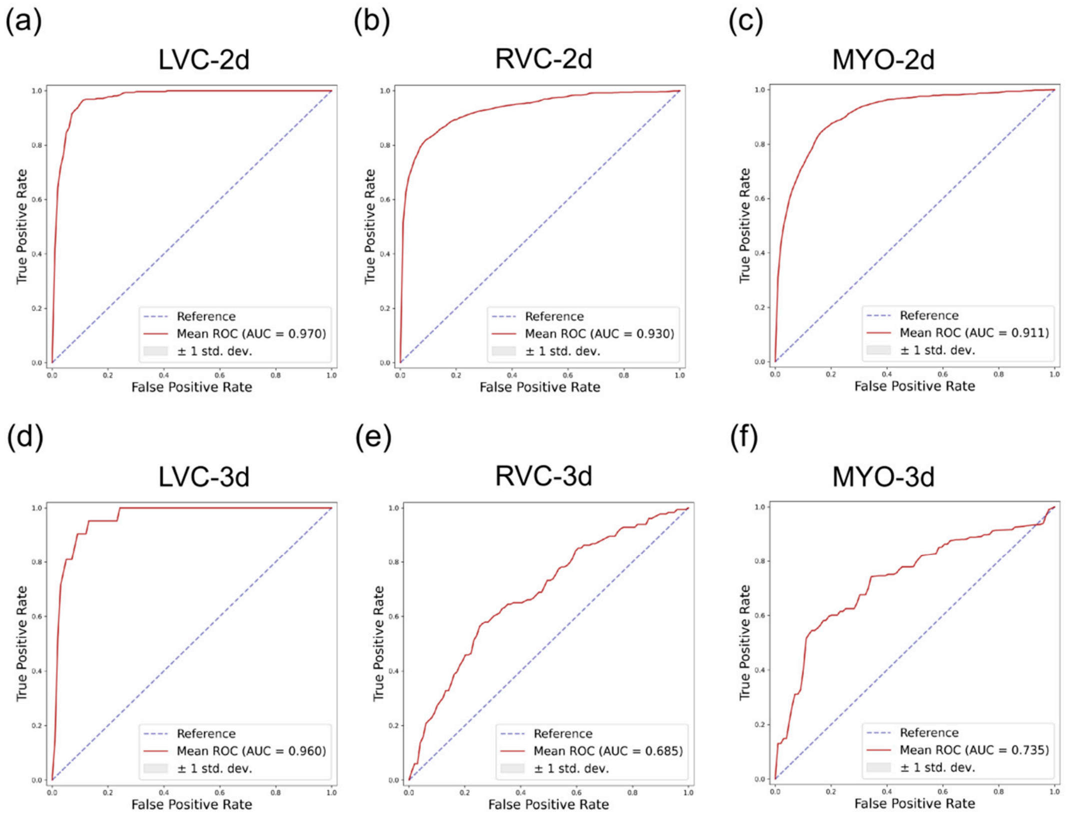

Supplementary Figure S2. ROC curves on external testing data among all six groups, the AUC and 95% confidence interval (CI) show on the figure legend. Subplots (a)-(f) are same as in supplementary Figure S1.

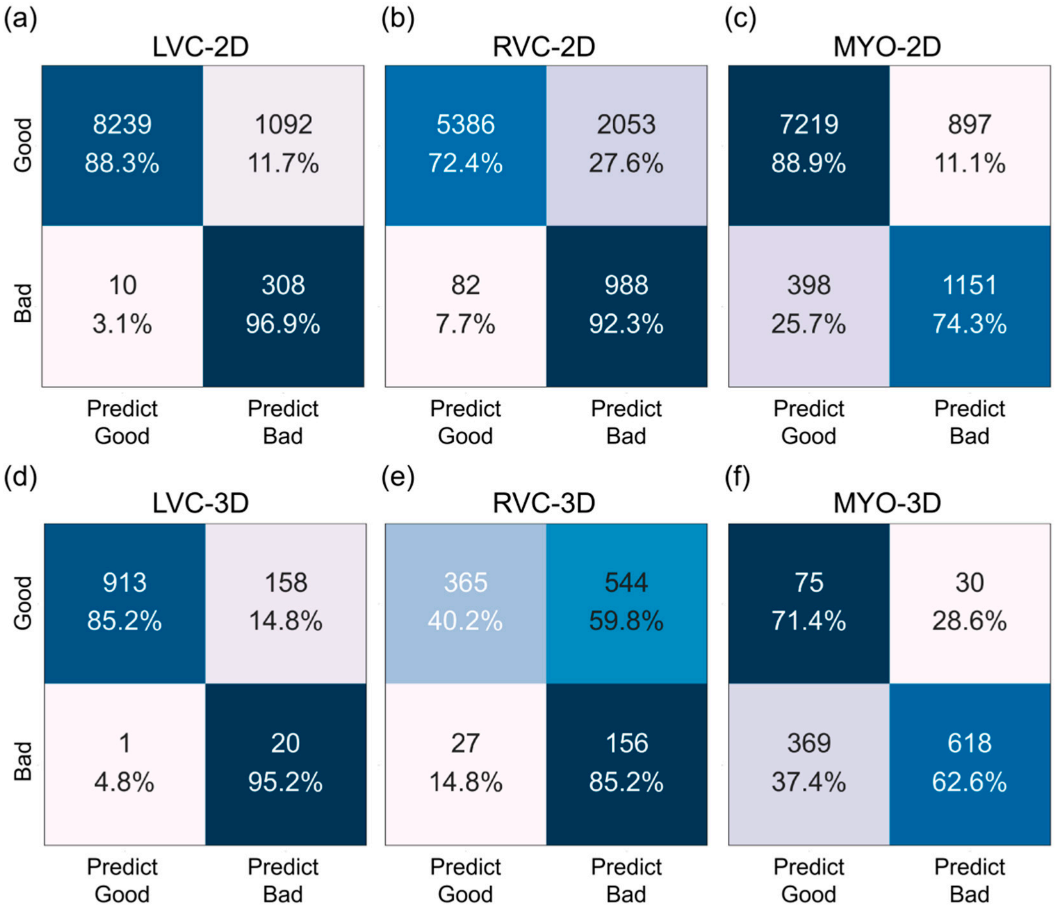

Supplementary Figure S3. Confusion matrices of best performance classification model among all six groups on external testing dataset. Subplots (a)-(f) are same as in supplementary Figure S1.

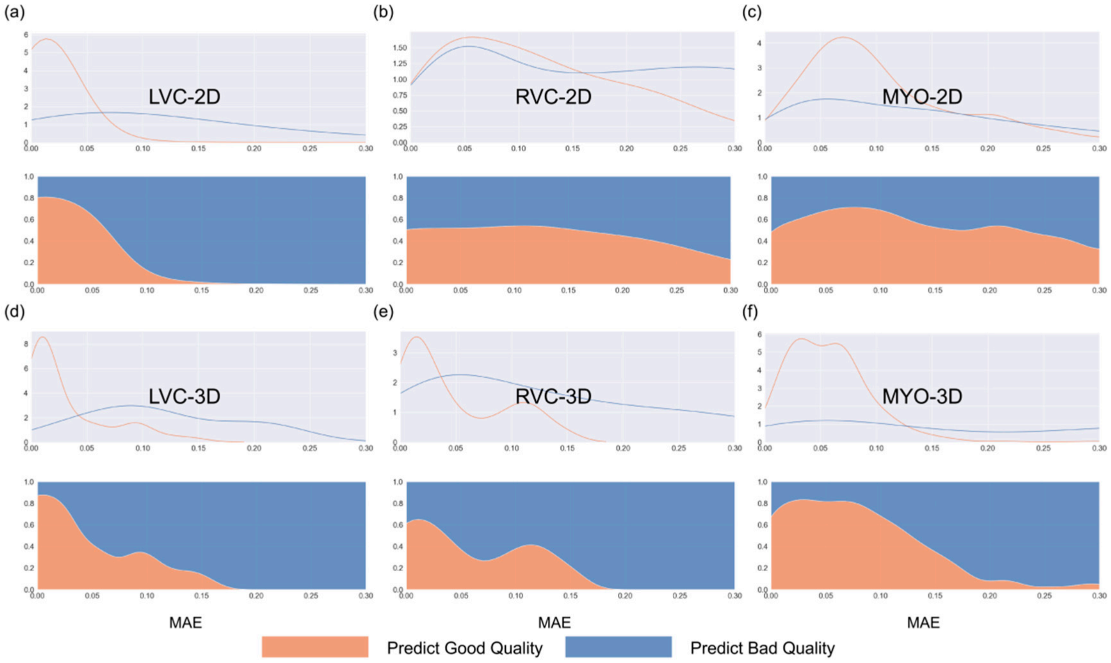

Supplementary Figure S4. Density plots for all segmentations among six groups on external testing dataset. Blue represents predicted bad quality and orange represents predicted good quality. The density plots in the first row represents the change between number of segmentations and MAE, while the density plots in the second row (filling mode) show the relative proportion of predicted good or bad segmentations with MAE. Subplots (a)-(f) are same as in supplementary Figure S1.

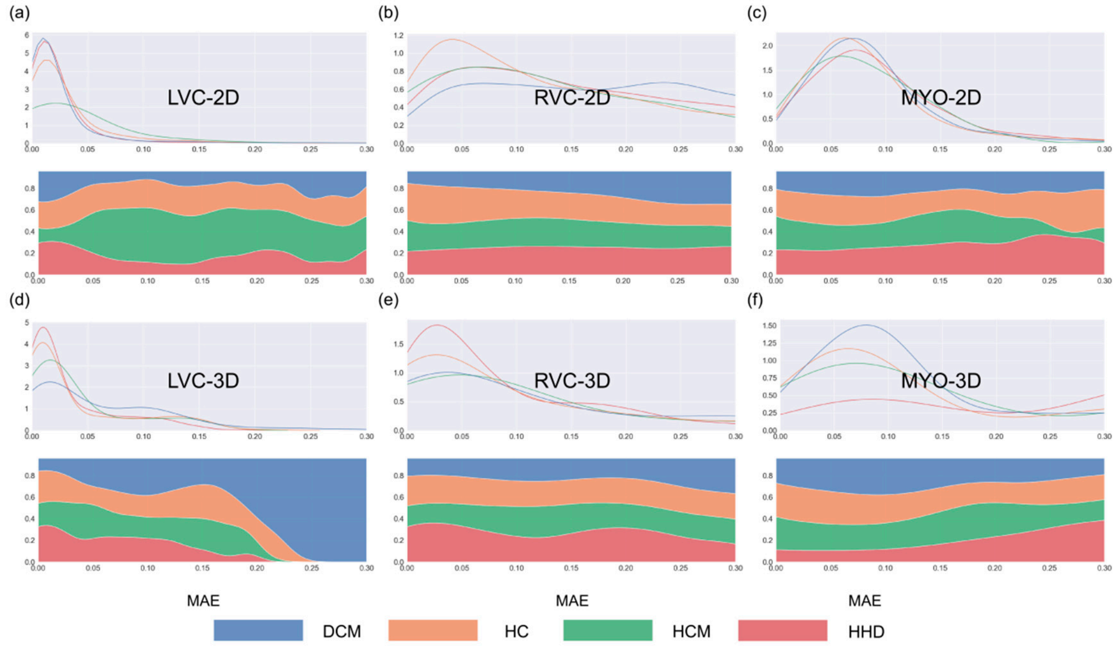

Supplementary Figure S5. Density plots for all segmentations among six groups on external testing dataset. Blue, orange, green and red represent DCM, HC, HCM and HHD, respectively. The density plots in the first row represents the change between number of segmentations and MAE, while the density plots in the second row (filling mode) show the relative proportion of different disease types with MAE. Subplots (a)-(f) are same as in supplementary Figure S1.

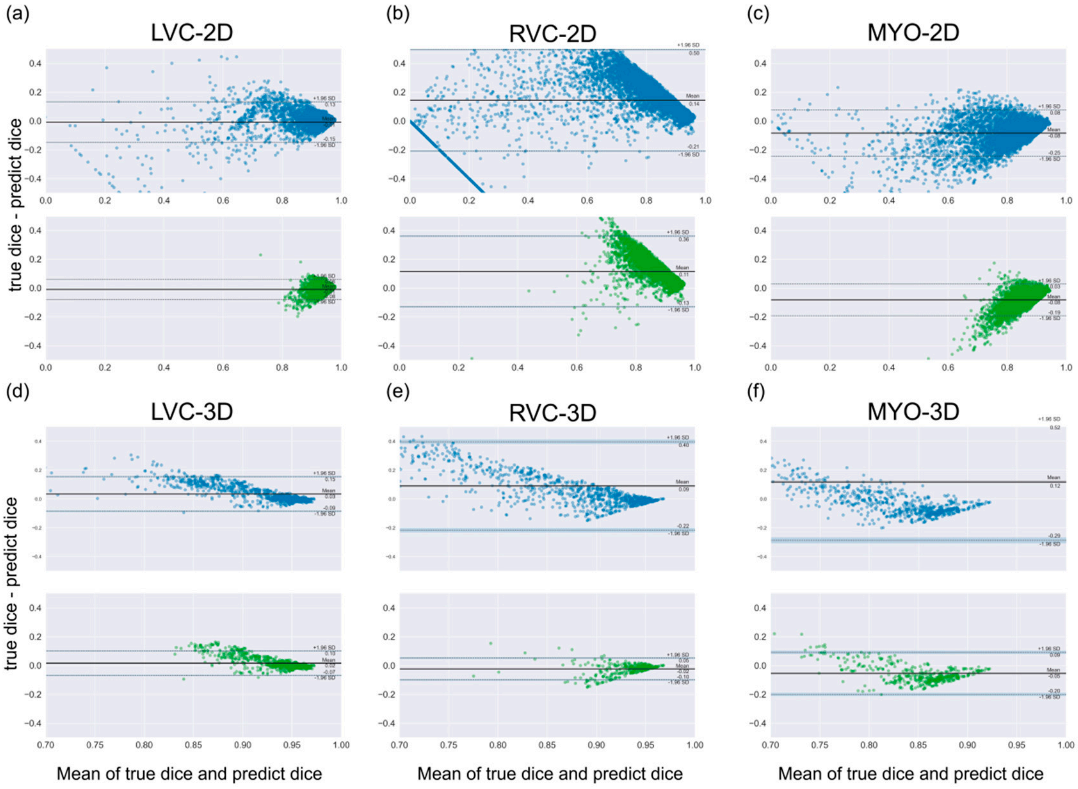

Supplementary Figure S6. Bland Altman plots of all segmentations (the first row with blue dots) and predicted good quality segmentations using classification models (the second row with green dots) in external testing dataset. Comparing two Bland Altman plots, the diminished blue dots represented segmentations that were predicted as bad quality. Subplots (a)-(f) are same as in supplementary Figure S1.

Supplementary Table S1. Shows the number of 2D and 3D segmentations for radiomics training dataset.

Training	LVC	RVC	MYO
2D	130738	118356	133207
3D	14874	14793	14793

Supplementary Table S2. Shows the number of 2D and 3D segmentations for radiomics testing dataset.

Testing	LVC	RVC	MYO
2D	4188	3662	4201
3D	460	460	460

Supplementary Table S3. Shows the full feature name list extracted in 3D images.

Shape	Elongation	GLCM	Autocorrelation
	Flatness		ClusterProminence
	LeastAxisLength		ClusterShade
	MajorAxisLength		ClusterTendency
	Maximum2DDiameterColumn		Contrast
	Maximum2DDiameterRow		Correlation
	Maximum2DDiameterSlice		DifferenceAverage
	Maximum3DDiameter		DifferenceEntropy
	MeshVolume		DifferenceVariance
	MinorAxisLength		Id
	Sphericity		Idm
	SurfaceArea		Idmn
	SurfaceVolumeRatio		Idn
	VoxelVolume		Imc1
Firstorder	10Percentile		Imc2
	90Percentile		InverseVariance
	Energy		JointAverage
	Entropy		JointEnergy
	InterquartileRange		JointEntropy
	Kurtosis		MCC
	Maximum		MaximumProbability
	MeanAbsoluteDeviation		SumAverage
	Mean		SumEntropy
	Median		SumSquares
	Minimum	GLDM	DependenceEntropy
	Range		DependenceNonUniformity
	RobustMeanAbsoluteDeviation		DependenceNonUniformityNormalized
	RootMeanSquared		DependenceVariance
	Skewness		GrayLevelNonUniformity
	TotalEnergy		GrayLevelVariance
	Uniformity		HighGrayLevelEmphasis
	Variance		LargeDependenceEmphasis
NGTDM	Busyness		LargeDependenceHighGrayLevelEmphasis
	Coarseness		LargeDependenceLowGrayLevelEmphasis
	Complexity		LowGrayLevelEmphasis
	Contrast		SmallDependenceEmphasis
	Strength		SmallDependenceHighGrayLevelEmphasis
GLSZM	GrayLevelNonUniformity		SmallDependenceLowGrayLevelEmphasis
	GrayLevelNonUniformityNormalized	GLRLM	GrayLevelNonUniformity
	GrayLevelVariance		GrayLevelNonUniformityNormalized
	HighGrayLevelZoneEmphasis		GrayLevelVariance
	LargeAreaEmphasis		HighGrayLevelRunEmphasis

	LargeAreaHighGrayLevelEmphasis		LongRunEmphasis
	LargeAreaLowGrayLevelEmphasis		LongRunHighGrayLevelEmphasis
	LowGrayLevelZoneEmphasis		LongRunLowGrayLevelEmphasis
	SizeZoneNonUniformity		LowGrayLevelRunEmphasis
	SizeZoneNonUniformityNormalized		RunEntropy
	SmallAreaEmphasis		RunLengthNonUniformity
	SmallAreaHighGrayLevelEmphasis		RunLengthNonUniformityNormalized
	SmallAreaLowGrayLevelEmphasis		RunPercentage
	ZoneEntropy		RunVariance
	ZonePercentage		ShortRunEmphasis
	ZoneVariance		ShortRunHighGrayLevelEmphasis
			ShortRunLowGrayLevelEmphasis

GLCM: gray level co-occurrence matrix; GLDM: gray level dependence matrix; GLRLM : gray level run length matrix; GLSZM: gray level size zone matrix; LOG: Laplace of Gaussian; NGTDM: Neighboring gray tone difference matrix.
Other abbreviation for specific features could be found in Pyradiomics documentation.

Supplementary Table S4. Shows the full feature name list extracted in2D images.

Shape	Elongation	GLDM	DependenceEntropy
	MajorAxisLength		DependenceNonUniformity
	MaximumDiameter		DependenceNonUniformityNormalized
	MeshSurface		DependenceVariance
	MinorAxisLength		GrayLevelNonUniformity
	Perimeter		GrayLevelVariance
	PerimeterSurfaceRatio		HighGrayLevelEmphasis
	PixelSurface		LargeDependenceEmphasis
	Sphericity		LargeDependenceHighGrayLevelEmphasis
Firstorder	10Percentile		LargeDependenceLowGrayLevelEmphasis
	90Percentile		LowGrayLevelEmphasis
	Energy		SmallDependenceEmphasis
	Entropy		SmallDependenceHighGrayLevelEmphasis
	InterquartileRange		SmallDependenceLowGrayLevelEmphasis
	Kurtosis	GLRLM	GrayLevelNonUniformity
	Maximum		GrayLevelNonUniformityNormalized
	MeanAbsoluteDeviation		GrayLevelVariance
	Mean		HighGrayLevelRunEmphasis
	Median		LongRunEmphasis
	Minimum		LongRunHighGrayLevelEmphasis
	Range		LongRunLowGrayLevelEmphasis
	RobustMeanAbsoluteDeviation		LowGrayLevelRunEmphasis
	RootMeanSquared		RunEntropy
	Skewness		RunLengthNonUniformity
	TotalEnergy		RunLengthNonUniformityNormalized
	Uniformity		RunPercentage
	Variance		RunVariance
NGTDM	Busyness		ShortRunEmphasis
	Coarseness		ShortRunHighGrayLevelEmphasis
	Complexity		ShortRunLowGrayLevelEmphasis
	Contrast	GLSZM	GrayLevelNonUniformity
	Strength		GrayLevelNonUniformityNormalized
GLCM	Autocorrelation		GrayLevelVariance
	ClusterProminence		HighGrayLevelZoneEmphasis
	ClusterShade		LargeAreaEmphasis
	ClusterTendency		LargeAreaHighGrayLevelEmphasis
	Contrast		LargeAreaLowGrayLevelEmphasis
	Correlation		LowGrayLevelZoneEmphasis
	DifferenceAverage		SizeZoneNonUniformity
	DifferenceEntropy		SizeZoneNonUniformityNormalized
	DifferenceVariance		SmallAreaEmphasis
	Id		SmallAreaHighGrayLevelEmphasis

	Idm		SmallAreaLowGrayLevelEmphasis
	Idmn		ZoneEntropy
	Idn		ZonePercentage
	Imc1		ZoneVariance
	Imc2		
	InverseVariance		
	JointAverage		
	JointEnergy		
	JointEntropy		
	MCC		
	MaximumProbability		
	SumAverage		
	SumEntropy		
	SumSquares		

GLCM: gray level co-occurrence matrix; GLDM: gray level dependence matrix; GLRLM : gray level run length matrix; GLSZM: gray level size zone matrix; LOG: Laplace of Gaussian; NGTDM: Neighboring gray tone difference matrix.

Other abbreviation for specific features could be found in Pyradiomics documentation.

Supplementary Table S5. Shows the best DSC prediction MAE of different structures with five regression models.

	Structures	Random Forest	Gradient Boost	K-Nearest Neighbour	Linear Regression	Multi-Layer Perceptron
2D	LVC	0.031	0.021	0.024	**0.020**	0.021
	RVC	0.071	**0.060**	0.069	0.075	0.059
	MYO	0.066	**0.032**	0.054	0.062	0.046
3D	LVC	0.013	**0.011**	0.013	0.016	0.014
	RVC	0.035	**0.027**	0.037	0.036	0.033
	MYO	0.020	**0.017**	0.020	0.030	0.023

Supplementary Table S6. Shows the best classification model performance of different structures with RF classifiers.

	Cardiac sub-structure	Accuracy	Precision	Recall	F1	NDR
2D	LVC	96.1	99.9	98.7	99.3	93.0
	RVC	95.9	99.1	92.9	95.9	90.0
	MYO	92.1	99.5	97.3	98.4	85.5
3D	LVC	99.1	100.0	99.8	99.9	100.0
	RVC	87.6	92.9	95.9	90.0	22.2
	MYO	89.1	94.9	86.1	90.3	75.0

Supplementary Table S7. Shows the top 12 features with highest mutual information score among 6 groups.

Structure	Feature name	Mutual Information Score
LVC-2D	**lvc_original_shape2D_PerimeterSurfaceRatio**	**0.404624174**
	**lvc_original_shape2D_Sphericity**	**0.383862508**
	**lvc_original_shape2D_MajorAxisLength**	**0.307065033**
	**lvc_original_firstorder_Energy**	**0.271998628**
	**lvc_original_shape2D_Elongation**	**0.248959322**
	**lvc_original_firstorder_90Percentile**	**0.162686545**
	lvc_original_gldm_LowGrayLevelEmphasis	0.16086934
	lvc_original_firstorder_Skewness	0.158071744
	lvc_original_firstorder_InterquartileRange	0.140505911
	lvc_original_glcm_ClusterShade	0.139599753
	lvc_original_gldm_LargeDependenceHighGrayLevelEmphasis	0.135106091
	lvc_original_firstorder_Minimum	0.132781633
RVC-2D	**rvc_original_shape2D_PerimeterSurfaceRatio**	**0.651769482**
	**rvc_original_shape2D_Sphericity**	**0.437640137**
	**rvc_original_shape2D_MeshSurface**	**0.42582423**
	**rvc_original_gldm_LowGrayLevelEmphasis**	**0.421417772**
	**rvc_original_firstorder_Minimum**	**0.395356937**
	**rvc_original_gldm_LargeDependenceLowGrayLevelEmphasis**	**0.30917076**
	**rvc_original_glszm_LargeAreaHighGrayLevelEmphasis**	**0.305207228**
	**rvc_original_firstorder_Entropy**	**0.264894479**
	**rvc_original_shape2D_MajorAxisLength**	**0.226848492**
	**rvc_original_firstorder_InterquartileRange**	**0.221537768**
	**rvc_original_shape2D_Elongation**	**0.20943546**
	**rvc_original_glszm_LargeAreaLowGrayLevelEmphasis**	**0.193302097**
MYO-2D	**myo_original_shape2D_Elongation**	**0.302787433**
	**myo_original_shape2D_PerimeterSurfaceRatio**	**0.29106965**
	**myo_original_shape2D_MeshSurface**	**0.21748549**
	**myo_original_shape2D_Sphericity**	**0.216930499**
	**myo_original_ngtdm_Strength**	**0.16516617**
	**myo_original_glszm_LargeAreaHighGrayLevelEmphasis**	**0.151299115**
	myo_original_ngtdm_Contrast	0.145962695
	myo_original_ngtdm_Busyness	0.140742989
	myo_original_firstorder_Energy	0.13926522
	myo_original_gldm_SmallDependenceLowGrayLevelEmphasis	0.134709171
	myo_original_firstorder_90Percentile	0.129108255
	myo_original_firstorder_Entropy	0.12807348
LVC-3D	**lvc_original_shape_Sphericity**	**0.801555464**
	**lvc_original_shape_SurfaceVolumeRatio**	**0.559563117**
	**lvc_original_shape_Maximum2DDiameterRow**	**0.401134873**
	**lvc_original_firstorder_Energy**	**0.291617701**
	**lvc_original_shape_Maximum2DDiameterSlice**	**0.228552073**

	**lvc_original_firstorder_90Percentile**	**0.225381944**
	**lvc_original_firstorder_Maximum**	**0.2157373**
	**lvc_original_ngtdm_Coarseness**	**0.20895502**
	**lvc_original_shape_MajorAxisLength**	**0.205803892**
	**lvc_original_firstorder_Minimum**	**0.205247547**
	**lvc_original_shape_Maximum2DDiameterColumn**	**0.203783679**
	**lvc_original_firstorder_Range**	**0.203574815**
RVC-3D	**rvc_original_shape_SurfaceVolumeRatio**	**0.953131487**
	**rvc_original_shape_Sphericity**	**0.760510463**
	**rvc_original_shape_MeshVolume**	**0.536610765**
	**rvc_original_firstorder_Energy**	**0.50027088**
	**rvc_original_ngtdm_Coarseness**	**0.499546949**
	**rvc_original_shape_SurfaceArea**	**0.37876214**
	**rvc_original_gldm_LargeDependenceHighGrayLevelEmphasis**	**0.37147254**
	rvc_original_firstorder_Range	0.356044341
	rvc_original_glcm_Autocorrelation	0.34359162
	rvc_original_glszm_LargeAreaEmphasis	0.341907675
	rvc_original_glcm_Contrast	0.334234088
	rvc_original_shape_MajorAxisLength	0.327433698
MYO-3D	**myo_original_ngtdm_Coarseness**	**0.71248084**
	**myo_original_shape_SurfaceVolumeRatio**	**0.576295938**
	**myo_original_shape_MeshVolume**	**0.575677495**
	**myo_original_ngtdm_Strength**	**0.551352337**
	**myo_original_firstorder_Energy**	**0.537937392**
	**myo_original_shape_Sphericity**	**0.347250649**
	**myo_original_gldm_LowGrayLevelEmphasis**	**0.329789701**
	**myo_original_glcm_Correlation**	**0.315698884**
	**myo_original_shape_MajorAxisLength**	**0.314293937**
	**myo_original_gldm_LargeDependenceLowGrayLevelEmphasis**	**0.312423599**
	**myo_original_shape_Maximum2DDiameterRow**	**0.302618722**
	myo_original_firstorder_Entropy	0.293561658
The bold lines represent features used in the best performance models.		

Supplementary Table S8. Shows the top 12 features with highest F-statistics among 6 groups.

Structure	Feature name	F-value	P-value
LVC-2D	**lvc_original_shape2D_Elongation**	**42844.52103**	**0**
	**lvc_original_shape2D_PerimeterSurfaceRatio**	**27573.03972**	**0**
	**lvc_original_shape2D_Sphericity**	**25123.83724**	**0**
	**lvc_original_firstorder_Entropy**	**13393.33004**	**0**
	**lvc_original_glcm_ClusterShade**	**6029.347552**	**0**
	**lvc_original_glcm_Imc1**	**5558.9091**	**0**
	lvc_original_firstorder_InterquartileRange	5201.938301	0
	lvc_original_gldm_LargeDependenceLowGrayLevelEmphasis	5036.970541	0
	lvc_original_firstorder_Minimum	4526.834322	0
	lvc_original_firstorder_Skewness	4152.646334	0
	lvc_original_gldm_LargeDependenceHighGrayLevelEmphasis	4097.109596	0
	lvc_original_shape2D_MajorAxisLength	3985.320238	0
RVC-2D	**rvc_original_firstorder_Minimum**	**71618.73185**	**0**
	**rvc_original_shape2D_PerimeterSurfaceRatio**	**63544.28013**	**0**
	**rvc_original_shape2D_Sphericity**	**50629.48021**	**0**
	**rvc_original_firstorder_Entropy**	**47690.18246**	**0**
	**rvc_original_shape2D_MeshSurface**	**47304.58763**	**0**
	**rvc_original_glcm_Imc1**	**23222.23486**	**0**
	**rvc_original_gldm_LowGrayLevelEmphasis**	**20117.45596**	**0**
	**rvc_original_firstorder_10Percentile**	**16475.38832**	**0**
	**rvc_original_glcm_Correlation**	**10142.88047**	**0**
	rvc_original_shape2D_Elongation	9493.814848	0
	rvc_original_glcm_ClusterShade	6588.276342	0
	rvc_original_ngtdm_Strength	6143.941558	0
MYO-2D	**myo_original_shape2D_Elongation**	**78701.22649**	**0**
	**myo_original_shape2D_PerimeterSurfaceRatio**	**28139.07476**	**0**
	**myo_original_ngtdm_Strength**	**15237.73372**	**0**
	**myo_original_ngtdm_Contrast**	**14561.11545**	**0**
	**myo_original_gldm_SmallDependenceLowGrayLevelEmphasis**	**10590.48295**	**0**
	**myo_original_firstorder_RootMeanSquared**	**10137.1095**	**0**
	**myo_original_glcm_Idmn**	**8763.984507**	**0**
	**myo_original_shape2D_MeshSurface**	**8385.514582**	**0**
	**myo_original_firstorder_90Percentile**	**8007.133039**	**0**
	myo_original_gldm_LowGrayLevelEmphasis	7136.382572	0
	myo_original_glcm_Imc1	5901.204943	0
	myo_original_ngtdm_Busyness	5785.002057	0
**LVC-3D**	**lvc_original_shape_Sphericity**	**19163.79911**	**0**
	**lvc_original_shape_Maximum2DDiameterRow**	**7908.196003**	**0**
	**lvc_original_shape_MajorAxisLength**	**2055.411936**	**0**
	**lvc_original_shape_Elongation**	**1241.62525**	**2.64E-261**
	**lvc_original_shape_Maximum2DDiameterSlice**	**958.0524434**	**6.49E-204**

	**lvc_original_shape_LeastAxisLength**	**761.1015916**	**1.95E-163**
	**lvc_original_glcm_ClusterShade**	**587.4403886**	**2.62E-127**
	**lvc_original_glcm_Idmn**	**461.3610504**	**8.22E-101**
	**lvc_original_shape_Maximum2DDiameterColumn**	**342.5007701**	**1.28E-75**
	**lvc_original_glcm_Imc1**	**268.7967322**	**6.95E-60**
	**lvc_original_shape_Flatness**	**226.9508617**	**6.54E-51**
	**lvc_original_ngtdm_Busyness**	**194.3892976**	**6.62E-44**
RVC-3D	**rvc_original_shape_Sphericity**	**24842.78218**	**0**
	**rvc_original_shape_SurfaceVolumeRatio**	**8701.631141**	**0**
	**rvc_original_glcm_Autocorrelation**	**5712.758803**	**0**
	**rvc_original_gldm_LargeDependenceHighGrayLevelEmphasis**	**5110.18352**	**0**
	**rvc_original_glszm_SizeZoneNonUniformityNormalized**	**4695.42225**	**0**
	rvc_original_glcm_Contrast	4168.090624	0
	rvc_original_shape_MajorAxisLength	4012.95452	0
	rvc_original_shape_MeshVolume	3320.261963	0
	rvc_original_firstorder_Range	3052.4025	0
	rvc_original_shape_Maximum2DDiameterSlice	2765.292385	0
	rvc_original_firstorder_Entropy	2724.8559	0
	rvc_original_firstorder_Energy	2428.71576	0
MYO-3D	**myo_original_shape_Elongation**	**3761.646652**	**0**
	**myo_original_shape_MeshVolume**	**3337.559842**	**0**
	**myo_original_firstorder_Entropy**	**2924.347996**	**0**
	**myo_original_shape_Maximum2DDiameterRow**	**2630.873004**	**0**
	**myo_original_gldm_LargeDependenceLowGrayLevelEmphasis**	**2224.323444**	**0**
	**myo_original_ngtdm_Contrast**	**2133.330609**	**0**
	**myo_original_shape_MajorAxisLength**	**1967.988222**	**0**
	**myo_original_glcm_Correlation**	**1917.750964**	**0**
	**myo_original_firstorder_Energy**	**1802.582054**	**0**
	**myo_original_shape_SurfaceVolumeRatio**	**1413.340411**	**1.72E-295**
	myo_original_firstorder_Skewness	1242.226744	2.27E-261
	myo_original_ngtdm_Strength	1008.304572	4.02E-214
The bold lines represent features used in the best performance models.			

Supplementary Table S9. Localization (whole heart segmentation) performance on independent external testing dataset.

Disease type	DSC	IoU
HCM	0.863	0.791
HHD	0.870	0.801
DCM	0.889	0.824
HC	0.865	0.794

Supplementary Table S10. RVC, LVC and MYO segmentation performance on independent external testing dataset.

Cardiac sub-structure	2D-DSC	3D-DSC
RVC	0.829	0.896
LVC	0.919	0.935
MYO	0.775	0.797

Supplementary Table S11. Classification model performance on independent external testing dataset with RF classifier.

	Cardiac sub-structure	Accuracy	Precision	Recall	F1	NDR
2D	LVC	88.6	99.9	96.4	98.1	96.9
	RVC	74.9	98.5	84.5	91.0	92.3
	MYO	86.6	94.8	86.2	90.3	74.3
3D	LVC	85.4	99.9	97.9	98.9	95.2
	RVC	47.7	93.1	70.1	80.0	85.2
	MYO	63.5	16.9	10.8	13.2	62.6

Supplementary Table S12. Regression model performance on independent external testing dataset with GB regressor.

	Cardiac sub-structure	ALL	DCM	HC	HCM	HHD
2D-DSC MAE	LVC	0.030	0.026	0.026	0.034	0.053
	RVC	0.183	0.209	0.185	0.147	0.169
	MYO	0.092	0.091	0.097	0.089	0.089
3D-DSC MAE	LVC	0.045	0.040	0.033	0.064	0.041
	RVC	0.116	0.113	0.084	0.147	0.129
	MYO	0.183	0.156	0.289	0.121	0.147

Supplementary Table S13. MAE improvement among different anatomical structures for all segmentations and predicted good quality segmentations.

	parameter	Cardiac sub-structure		
		RVC	MYO	LVC
2D	MAE improvement	0.052	0.008	0.012
	P-value	0.231	0.847	0.521
3D	MAE improvement	0.085	0.103	0.016
	P-value	<0.001	<0.001	<0.001
